# Cryopreservation of assay-ready hepatocyte monolayers by chemically-induced ice nucleation: preservation of hepatic function and hepatotoxicity screening capabilities†

**DOI:** 10.1039/d3bm01046e

**Published:** 2023-10-16

**Authors:** Ruben M. F. Tomás, Robert Dallman, Thomas R. Congdon, Matthew I. Gibson

**Affiliations:** a Division of Biomedical Sciences, Warwick Medical School, University of Warwick Gibbet Hill Road Coventry CV4 7AL UK m.i.gibson@warwick.ac.uk; b Department of Chemistry, University of Warwick Gibbet Hill Road Coventry CV4 7AL UK; c Cryologyx Ltd 71-75 Shelton Street London WC2H 9JQ UK

## Abstract

Cell culture plays a critical role in biomedical discovery and drug development. Primary hepatocytes and hepatocyte-derived cell lines are especially important cellular models for drug discovery and development. To enable high-throughput screening and ensure consistent cell phenotypes, there is a need for practical and efficient cryopreservation methods for hepatocyte-derived cell lines and primary hepatocytes in an assay-ready format. Cryopreservation of cells as adherent monolayers in 96-well plates presents unique challenges due to low volumes being susceptible to supercooling, leading to low recovery and well-to-well variation. Primary cell cryopreservation is also particularly challenging due to the loss of cell viability and function. In this study, we demonstrate the use of soluble ice nucleator materials (IN) to cryopreserve a hepatic-derived cell line (HepG2) and primary mouse hepatocytes, as adherent monolayers. HepG2 cell recovery was near 100% and ∼75% of primary hepatocytes were recovered 24 hours post-thaw compared to just 10% and 50% with standard 10% DMSO, respectively. Post-thaw assessment showed that cryopreserved HepG2 cells retain membrane integrity, metabolic activity, proliferative capacity and differentiated hepatic functions including urea secretion, cytochrome P450 levels and lipid droplet accumulation. Cryopreserved primary hepatocytes exhibited reduced hepatic functions compared to fresh hepatocytes, but functional levels were similar to commercial suspension-cryopreserved hepatocytes, with the added benefit of being stored in an assay-ready format. In addition, normal cuboidal morphology and minimal membrane damage were observed 24 hours post-thaw. Cryopreserved HepG2 and mouse hepatocytes treated with a panel of pharmaceutically active compounds produced near-identical dose–response curves and EC_50_ values compared to fresh hepatocytes, confirming the utility of cryopreserved bankable cells in drug metabolism and hepatotoxicity studies. Cryopreserved adherent HepG2 cells and primary hepatocytes in 96 well plates can significantly reduce the time and resource burden associated with routine cell culture and increases the efficiency and productivity of high-throughput drug screening assays.

## Abstract 2

Controlled ice nucleation enables cryopreservation of primary hepatocytes monolayers, in assay ready format.

## Graphical abstract

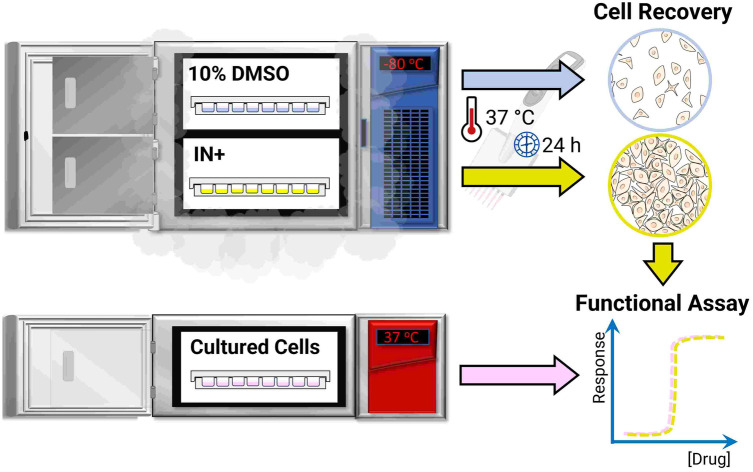

## Introduction

Cell culture is a platform technology spanning biomedical discovery science to pharmaceutical and biotechnology industries. Accurate and reproducible prediction of drug efficacy and safety is crucial for new drug candidates to enter Phase I clinical trials, requiring the understanding of drug metabolism pathways, potential drug–drug interactions, and adverse effects.^1^ As the liver is the primary site for drug metabolism, primary hepatocytes and the HepG2 cell line, a hepatocellular carcinoma-derived cell line exhibiting many differentiated hepatic functions, are the most widely used *in vitro* models in pharmacological and toxicological studies to screen drugs for hepatotoxicity, cytochrome P450 induction/inhibition, and potential drug–drug interactions.^2,3^ Cryopreservation of cells in suspension (vials) is the current approach for long-term storage and transportation, but most laboratory-based drug screening applications require cells as adherent monolayers.^4^ Thus, cryopreserved cell lines must be thawed from suspension, propagated, and finally plated into well plates for drug screening applications, which takes significant time and resources. The routine handling of cell lines also generates multiple types of single-use plastic waste (with different disposal/recycling routes), the potential for genetic drift and requires specialist knowledge (*e.g.* substrate specificity, growth factors, inducers) to establish.^5–7^ Specifically, cryopreserved primary hepatocytes exhibit reduced hepatic functions compared to fresh, such as albumin and urea secretion, and lower initial attachment efficiency due to the downregulation of adhesion proteins.^8–11^ This poses a challenge as high-density hepatocyte monolayers are necessary to ensure high viability.^12^ Therefore, a practical, efficient, and sustainable method for the cryopreservation of hepatocyte-derived cell lines and primary hepatocytes in an assay-ready format, such as monolayers in well plates, would facilitate drug metabolism studies and high-throughput screening. The distribution of assay-ready cells would also ensure phenotypically identical cells and save days/weeks of cell culture, optimising time spent by cell users.

The gold standard cryoprotectant for mammalian cell cryopreservation in suspension is 10% dimethyl sulfoxide (DMSO). However, this cryoprotectant is unable to cryopreserve adherent cell monolayers,^13–15^ which is likely due to fatal intracellular ice propagation from cell–cell contacts.^16–18^ For a successful cryopreservation outcome, cells should be cooled fast enough to minimise the exposure time to potentially cytotoxic cryoprotectants and increased solute concentrations during ice formation, but slow enough to allow cells to dehydrate and reduce the probability of fatal intracellular ice formation (IIF).^19^ Natural and synthetic polymers, such as antifreeze proteins and polyproline,^4,20,21^ can improve post-thaw outcomes by inhibiting ice recrystallization or stabilising cell membranes during freezing. Preconditioning of HepG2 cells and other cell lines with trehalose and proline has also been shown to increase post-thaw cell recovery from approximately 10–20% to 50%.^22,23^ However, this recovery rate is insufficient for assay-ready banking. Polyampholytes, polymers bearing mixed anionic/cationic side chains, have been demonstrated to have potent cryoprotective properties. Carboxylated poly(ε-lysine) (PLL) and 10% (v/v) DMSO performed twice as well as DMSO alone for the freezing of rat bone marrow mesenchymal stem cells and L929 as monolayers.^24^ Polyampholyte's mechanism of cryoinjury protection is unclear, although evidence suggests that a matrix is formed surrounding cells to trap salts and minimize osmotic damage, whilst also minimising deleterious intracellular ice formation by allowing sufficient cellular dehydration.^25–27^ Polyampholytes may also interact/protect the cell membrane, similar to how antifreeze proteins can protect liposome models.^24,28^ DMSO is still required in combination with the above cryoprotectants as it replaces lost water to prevent excessive dehydration and minimise osmotic shock from reduced electrolyte concentration in unfrozen solutions during freezing.^29,30^ Bailey *et al.* introduced a synthetically scalable polyampholyte based on the ring opening of poly(methyl vinyl ether-*alt*-maleic anhydride) to cryopreserve a panel of cell lines as adherent monolayers in 24-well plates.^15^ Subsequent studies expanded on this and demonstrated that A549, HepG2 and Caco-2 cells cryopreserved as monolayers in 24-well plates were “Assay Ready” 24 hours post-thaw.^13^ Over 80% of cells were recovered with preserved healthy morphology, intact membranes, minimal apoptosis, compatibility with multiple drug screening assays, and retained normal proliferative capacity and metabolic activity. However, the above cryopreservation examples have been limited to 24-well plates, which are useful for many assays but may not be suitable for high-throughput analysis.

Cryopreservation of cells in 96 well plates, as monolayers, would be more desirable for high-throughput screening applications but the transition from 24 to 96 well plates is not trivial. The low volumes in 96 well plates (∼100 μL) are more likely to supercool than larger volumes, resulting in the cryopreservation medium not freezing until ∼−20 °C. Delayed freezing prevents cellular dehydration, as thermodynamically stable extracellular ice is required to increase osmolarity (due to ice forming a pure phase) and promote water transfer, resulting in low cell recovery values.^31^ Moreover, ice formation is an exothermic process that releases more heat during supercooling, which can lead to local thawing and influences cooling rates. As ice nucleation is stochastic, high variability in the freezing and heat of recrystallisation is observed across a 96 well plate.^32^ Therefore, methods used for monolayer cryopreservation in well plates with larger volumes are not always useful for 96 well plate format, where inducing nucleation at sub-zero temperatures is essential.^32,33^ Controlled ice nucleation for cell cryopreservation has been achieved by mechanical (strong electric fields^34^ and directional freezing)^35^ and chemical (pollen washing water,^36^ cholesterol monohydrate,^37,38^ Snowmax™,^39^ sand,^40^ ice mist^32^ and biologically inert minerals)^41^ methods. Pollen washing water (PWW) is particularly interesting as it is the only water-soluble, easily removable, predictable chemical ice nucleator, produced by suspending Hornbeam (*Carpinus betulus*) pollen grains in water.^36^ The component allowing controlled nucleation is believed to be a carboxylic acid-bearing polysaccharide of mass greater than 100 kDa (ref. 42) and has been demonstrated to aid in the cryopreservation of A549 and HepG2 cells in 96 well plates as confluent monolayers.^43^ Despite the reported high cell recovery rates, the study did not have an in-depth examination of crucial factors that are important for the practicality of utilising cryopreserved cell monolayers in drug screening applications. These factors include well-to-well variability, viability and metabolic assessments, as well as hepatotoxicity challenges. Furthermore, ice nucleators have yet to be explored for the cryopreservation of primary hepatocyte monolayers, where current cryoprotectants employed have proven to be ineffective.^23,44,45^

Herein we induced ice nucleation (IN) for the successful and reproducible cryopreservation of HepG2 cells and primary mouse hepatocytes as monolayers in 96 well plates, enabling their direct use in toxicological screening 24 hours post-thaw (“assay ready”). The cryopreservation process yielded very encouraging results, with nearly 100% recovery of HepG2 cells, approximately 75% recovery of primary hepatocytes and minimal well-to-well variation post-thaw. Cryopreservation of HepG2 cells using DMSO alone, cholesterol monohydrate (as an alternative nucleator), and a polyampholyte, was attempted but lower recoveries were obtained. Post-thaw analysis revealed that HepG2 cells retained normal proliferative capacity and hepatic functions, including urea secretion, CYP450 levels, and lipid droplet accumulation in response to free fatty acid solutions, confirming their assay readiness. Primary hepatocytes also preserved their cellular morphology and showed less than a 5% increase in membrane damage post-thaw. Although a reduction in primary hepatocyte function was observedthese levels remained comparable to commercial suspension-cryopreserved hepatocytes, with the added advantage of being readily available in an assay-ready format. Cryopreserved HepG2 cells and mouse hepatocytes treated with a panel of pharmaceutically active compounds yielded near-identical dose–response curves and EC_50_ values compared to non-frozen cells, affirming the utility of cryopreserved bankable cells in drug metabolism and hepatotoxicity studies.

## Results and discussion

The primary aim of this study was to cryopreserve HepG2 cells and primary mouse hepatocytes as adherent cell monolayers in 96 well plates (an “assay ready” format) while maintaining their normal cellular viability and differentiated hepatic functions 24 hours after thawing. By achieving this, the need for routine cell culture can be eliminated, as users would only require a thawing step, enabling rapid and barrier-free drug screening and toxicological studies with minimal user effort. Current cryopreservation methods are unable to freeze cells in 96 well plates, due to the cold-induced damage caused by the supercooling of water at volumes <100 μL to temperatures as low as −20 °C.^36,43^ Furthermore, the stochastic nature of nucleation also results in high well-to-well variability without controlled nucleation. A panel of cryoprotectants were initially screened using HepG2 cells to determine which would provide the best post-thaw cell recovery including 10% DMSO and 10% DMSO supplemented with polyampholyte (demonstrated to be potent in larger volume 24-well plates),^13^ cholesterol monohydrate crystals^37,38^ or Hornbeam pollen washing water (PWW); data can be found in the ESI (Fig. S1 and S2†). As expected, cell recovery using 10% DMSO was low (12.6%) and was improved with the addition of polyampholyte (<30%). Significant increases were observed with the addition of the ice nucleators cholesterol monohydrate and PWW, increasing cell recoveries to ∼60% and ∼90%, respectively, affirming the importance of ice nucleators in the cryopreservation of cell monolayers in low volumes, by preventing supercooling.

**Fig. 1:**
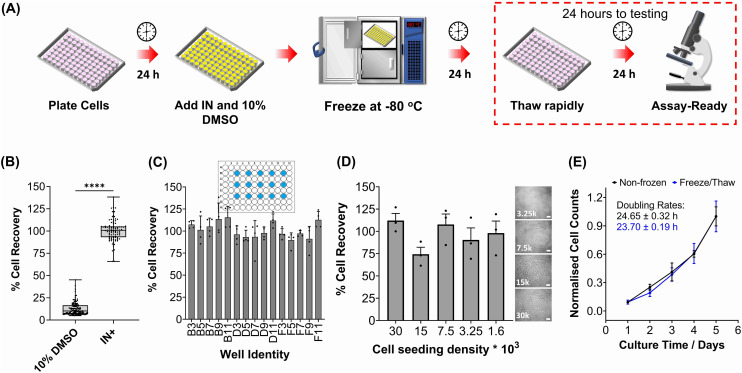
HepG2 cell monolayer recovery post-thaw. (A) Cryopreservation workflow to produce “Assay Ready” HepG2 96 well plates. HepG2 cells were cryopreserved using 10% DMSO plus an ice nucleator (IN) as the cryoprotectant. Post-thaw percentage cell recovery of HepG2 confluent monolayers was reported as (B) an overall average (10% DMSO control included) ± upper/lower quartiles and the min/max value of 4 biological repeats and 24 technical repeats (Welch *T*-test; ****p ≤ 0.0001) and (C) individual well results ± SEM of 4 biological repeats. (D) Post-thaw percentage cell recovery of HepG2 cells plated at different densities ± SEM of 3 biological repeats with post-thaw phase contrast images provided (scale bar = 100 μm). Enlarged images can be found in the ESI.† (E) Growth curves of non-frozen and freeze/thaw HepG2 cells produced by daily cell counts which were normalised (± SEM of 3 biological repeats) to calculate the proliferation rate.

PWW was selected for the cryopreservation of hepatic cells due to the higher cell recoveries obtained and the water-soluble nature of the nucleator, which simplifies the process of addition and removal compared to other nucleators such as cholesterol monohydrate and feldspar.^46^ The workflow to produce “assay ready” HepG2 cells in 96 well plates is outlined in Fig. 1A. Briefly, HepG2 cells are seeded for 24 hours, the cell culture medium is replaced with the IN and 10% DMSO and the plates are placed immediately in a −80 °C freezer where they can be stored until required. Rapid freezing in this manner is thought to promote innocuous IIF.^47^ The cryopreserved HepG2 plates are thawed rapidly with warm cell culture medium, to minimise cellular damage from intracellular ice reformation,^48^ and are ready-to-use in 24 hours. All experiments were conducted 24 hours post-thaw to allow programmed cell death pathways to complete, which take 12–24 hours,^49^ thus removing the risks of exaggerating post-thaw cell recovery and viability that can lead to false positive results.^50^ Additional repeats revealed that total post-thaw cell recovery of HepG2 cells (100%) was achieved through the use of PWW and 10% DMSO, whereas only 10% of cells were recovered with 10% DMSO alone, Fig. 1B. Individual cell recovery values can be above 100% due to post-thaw proliferation of cells. Preconditioning HepG2 cells with trehalose only allows cell recoveries to reach 42%,^23^ so this is a considerable improvement over existing technologies. Post-thaw cell recovery values were also provided for individual wells to highlight any variations across the well plate, a parameter previously unexplored that would impact assay results in drug screening applications, Fig. 1C (data on additional wells can be found in the ESI†). Minimal well-to-well variation was observed. Moreover, HepG2 cells were successfully cryopreserved at different cell densities (1.6–30 k cells per well), Fig. 1D, with only small changes to cell recovery values observed (no significant trend observed). Phase contrast imaging illustrated no changes to cellular morphology between non-frozen and freeze/thaw HepG2 cells regardless of seeding density (enlarged images can be found in the ESI, Fig. S6†). Successfully cryopreserving cells at different seeding is crucial for assay development and toxicological screening. For example, cell viability assays require a linear response between cell seeding density and the signal output (usually absorbance or fluorescence), to infer cell viability when testing pharmaceutically active compounds.

HepG2 cells were counted daily following thawing to determine their proliferative capacity. The growth curves produced for non-frozen and freeze/thaw HepG2 cells were nearly identical, along with their doubling rate, Fig. 1E. Short-term storage (<1 month) of suspension cryopreserved cells at −80 °C does not alter post-thaw proliferation rate^51,52^ and the same is expected for adherent cells stored under the same conditions, based on these findings. A549 cells were also cryopreserved using the same workflow to determine its compatibility with an additional cell line (Fig. S3–S6†). Percentage cell recovery was 98% and unaffected by cell seeding density, compared to 29% with 10% DMSO alone, and doubling rates were comparable to non-frozen cells. Storage of both A549 and HepG2 cells for over 1 month had no impact on cell recovery values, Fig. S7.†

**Fig. 2:**
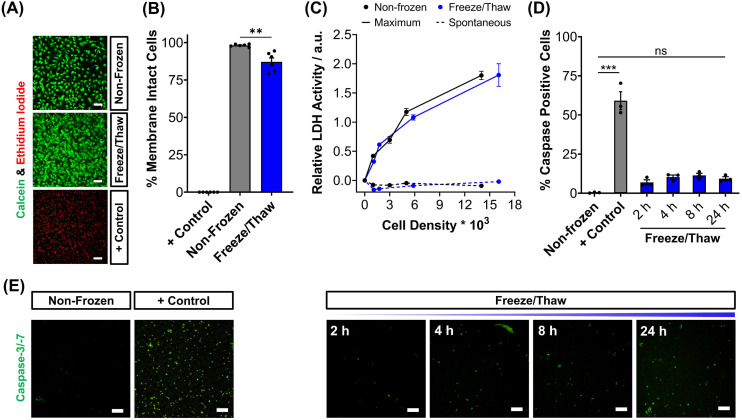
Post-thaw HepG2 viability assessment. (A) Sample fluorescence images of non-frozen, freeze/thaw and methanol-treated (70%, + control) HepG2 cells stained with calcein (green, membrane intact) and ethidium iodide (red, membrane damaged). (B) The percentage of membrane intact cells ± SEM of 3 biological and 2 technical repeats was calculated from the fluorescence images of cells stained with calcein and ethidium iodide. (C) The total lactate dehydrogenase (LDH) activity (solid line, maximum) found in non-frozen (black) and freeze/thaw (blue) HepG2 cells was measured and the activity of LDH released into cell culture medium (dashed, spontaneous) ± SEM of 3 biological and 2 technical repeats. (D) Percentage of caspase-3/-7 positive cells in non-frozen, freeze/thaw and staurosporine-treated cells (2 μM, + control) ± SEM of 3 biological. (E) Sample fluorescence images of real-time caspase-3/-7 (green) activation in freeze/thaw HepG2 cells and of non-frozen and staurosporine-treated cells (2 μM, + control) after 24 hours. Scale bars = 100 μm. (ANOVA, Tukey PostHoc; ns: *p* ≥ 0.05, **p ≤ 0.01, ***p ≤ 0.001).

To validate the efficacy of this cryopreservation technology, it was essential to assess the viability of HepG2 cells using a range of functional assays, focusing on those commonly employed to determine EC_50_ values of pharmaceutically active compounds during drug screening. Non-frozen and freeze/thaw HepG2 cells were stained with calcein (green, membrane intact) and ethidium iodide (EI, red, membrane intact) to visualise membrane damage caused by the freezing process, Fig. 2A. Minimal membrane damaged cells were observed in the freeze/thaw samples. The percentage of cells with intact membranes was quantified by calculating the ratio of calcein-positive cells to the total cell count, Fig. 2B. A small decrease in the proportion of cells with intact membranes was observed after freeze/thaw, from 98% to 87%, which is consistent with our previous results from 24-well plate freezing with polyampholyte and is sufficiently small not to impact performance.^13^

The release of lactate dehydrogenase (LDH) into cell culture medium (spontaneous release) and the total LDH intracellular content of HepG2 cells (maximum) was also measured before and after freezing as an additional measure of membrane intactness, Fig. 2C. LDH is an enzyme with a molecular weight over 140 kDa,^53^ so large defects in the membrane are required for its release and may suggest that other crucial enzymes required for Phase I/II metabolism of drugs could also be released. No difference was observed between the intra- and extra-cellular LDH content of non-frozen and freeze/thaw HepG2 cells confirming that physical disruption of cells is largely avoided during cryopreservation with IN, reducing risks of necrotic cell death pathways. The LDH assay is a standard tool in drug discovery to monitor apoptotic and necrotic processes so these findings are crucial in proving the compatibility and “assay ready” nature of freeze/thaw HepG2 cells 24 hours post-thaw.^54^

The activation of intrinsic, extrinsic and calpain programmed cell death pathways during cryopreservation has led to an increased interest in supplementing apoptotic inhibitors into cryoprotectant formulations or post-thaw medium to improve cell recovery.^55,56^ The dominant mechanism for apoptosis activation in hepatocyte cryopreservation is mitochondrial membrane potential disruption by oxidative stress (from reactive oxygen species), causing cytochrome C release and the translocation of P53, which initiates the caspase cascade pathway.^57–59^ Given the significance of apoptosis in cryopreservation outcomes, the induction of executioner caspases-3 and -7 (cysteinyl aspartate-specific proteases) was monitored over time for freeze/thaw cells and compared to non-frozen cells treated with and without staurosporine, a highly potent inductor of apoptosis by inhibiting kinases, Fig. 2D. Images of caspase-3/-7 activation have also been provided, Fig. 2E. Although caspase activation increased from 0.4% to 9%, confirming an increase in programmed cell death, this is still an impressive outcome. For comparison, mesenchymal stem cells typically exhibit apoptosis levels of 40% post-thaw, hence the need to explore apoptotic inhibitors, whereas our system would likely have minimal benefits. A549 cells also displayed minimal decreases in percentage membrane intact cells (from 99% to 94%) and increases in caspase activation (from 1% to 10%) post-thaw, Fig. S9–S13.†

**Fig. 3:**
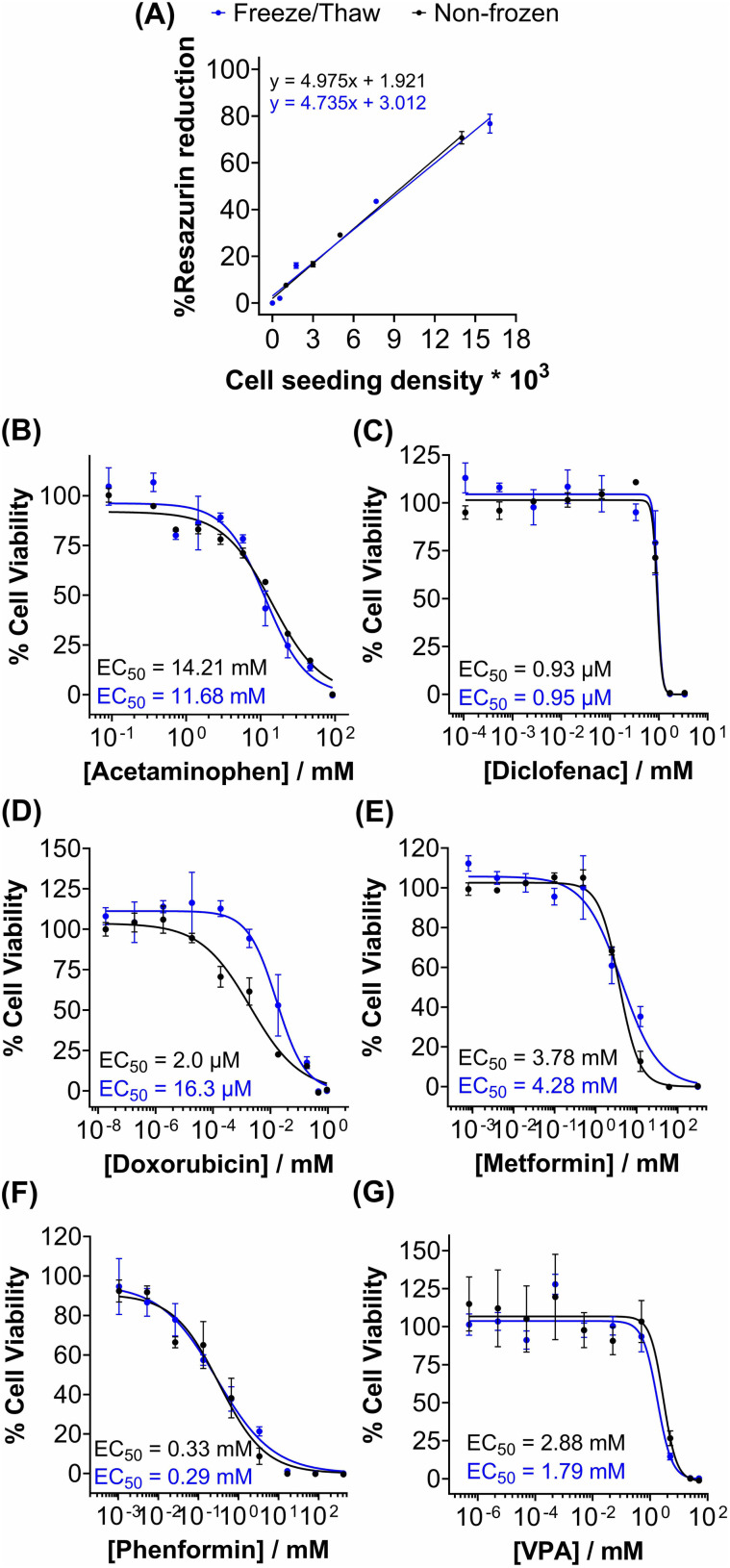
Rapid drug screening with freeze/thaw HepG2 cells. (A) Percentage resazurin reduction of non-frozen (black) and freeze/thaw (blue) HepG2 cells plated at different cell densities ± SEM of 3 biological repeats and 2 technical repeats. Non-frozen (black) and freeze/thaw (blue) HepG2 cells were treated with (B) acetaminophen (0–92.6 mM), (C) diclofenac (0–3.4 mM), (D) doxorubicin (0–9.2 mM), (E) metformin (0–310 mM), (F) phenformin (0–413 mM) and (G) valproic acid (0–50 mM) for 24 h and a resazurin reduction assay was used to measure % cell viability ± SEM of 3 biological repeats and 2 technical repeats to determine EC_50_ values.

Metabolic activity and ATP-based assays are crucial indicators of cell viability and are commonly used to screen drug candidates. The metabolic activity of freeze/thaw HepG2 cells was measured using a resazurin reduction assay, Fig. 3A. Direct proportionality was observed between the quantity of HepG2 cells cryopreserved and percentage cell viability, confirming that the resazurin reduction assay can be used to determine EC_50_ values in drug screening. Moreover, there were no differences in the metabolic activity of non-frozen and freeze/thaw HepG2 cells 24 hours post-thaw. Non-frozen and cryopreserved HepG2 cells were subsequently treated with clinically relevant pharmaceutically active compounds that are heavily metabolised by CYP enzymes and have been previously used in the development of 3-D HepG2 and hepatocyte models to study drug-induced liver injury.^60–62^ Cell viability was measured using the resazurin reduction assay to plot dose–response curves and determine drug EC_50_ values, Fig. 3B–G. Minimal differences were found between the dose–response curves and EC_50_ values of the drug candidates tested against non-frozen and freeze/thaw HepG2 cells. Therefore, freeze/thawed HepG2 cells are both “assay-ready” and capable of providing a faster, simpler, and more cost-effective method for screening drug compounds, without increasing susceptibility to cell death caused by the cryopreservation process.

**Fig. 4:**
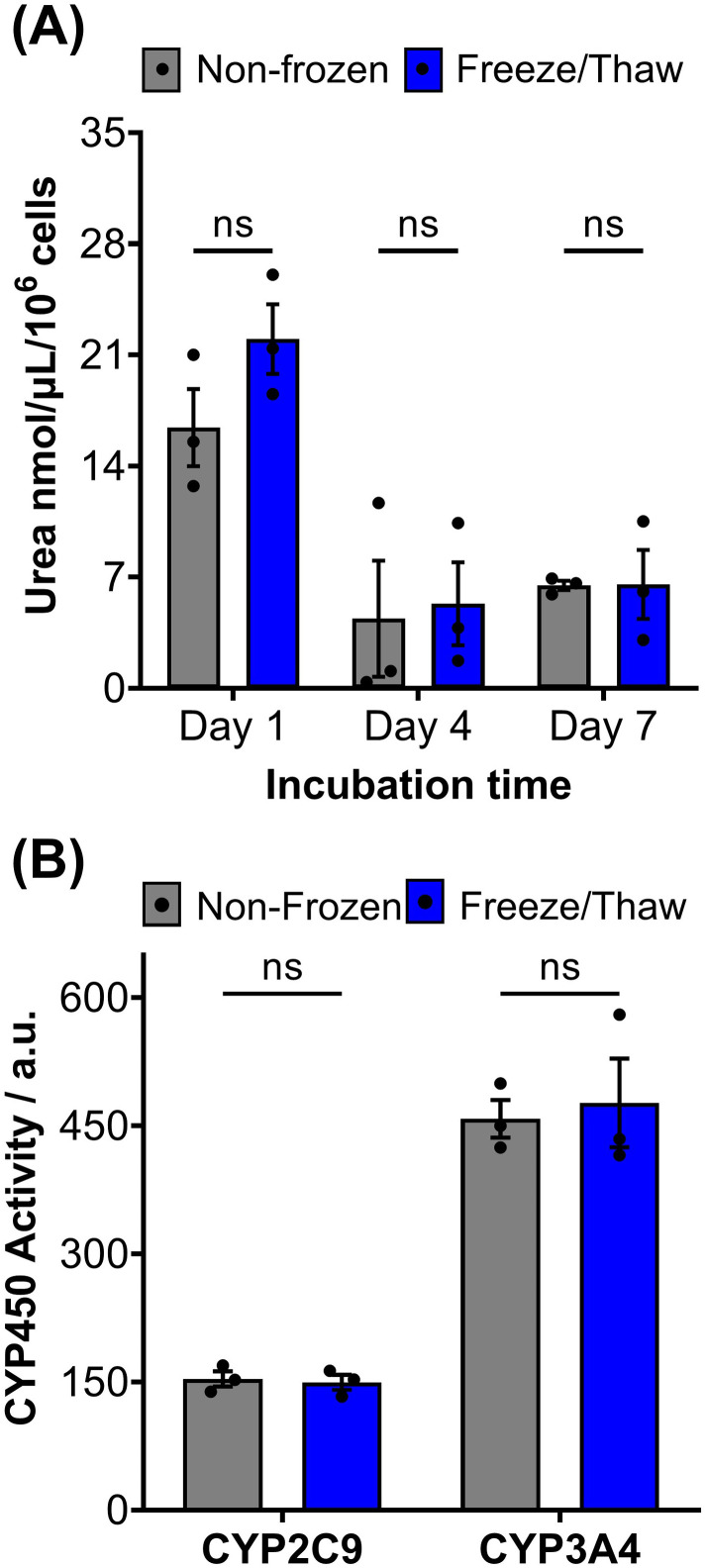
HepG2 differentiated hepatic functions are retained post-thaw. (A) Urea secretion of non-frozen (grey) and freeze/thaw (blue) HepG2 cells after 1, 4 and 7 days of culture. (B) CYP2C9 and CYP3A4 basal activity of non-frozen (grey) and freeze/thaw (blue) HepG2 cells 24 hours post-thaw. Three biological repeats were completed (ANOVA, Tukey PostHoc ns: *p* ≥ 0.05).

HepG2 cells exhibit a range of differentiated hepatic functions, making them a valuable cell line for investigating drug metabolism, which serve as additional indicators for evaluating the effectiveness of our cryopreservation approach.^62^ The urea cycle, an essential detoxification pathway responsible for converting ammonia to urea, serves as a sensitive indicator of hepatic function and can be influenced by factors such as pharmacologically active substances, *in vitro* environmental conditions, and genetic modifications.^63–65^ Evaluation of freeze/thaw HepG2 cells revealed no significant alterations in urea secretion per cell (Fig. 4A) or urea accumulation in the culture medium over 7 days (Fig. S17†), indicating the preservation of urea synthesis capacity. The post-thaw activity of cytochrome P450 (CYP) enzymes was also assessed given their essential role in understanding drug–drug interactions (CYP induction/inhibition), drug clearance, metabolite formation, and hepatotoxicity detection in drug screening applications. CYP3A4, in particular, is responsible for metabolizing more than 50% of pharmaceutically active compounds.^66^ No significant differences were found in the CYP3A4 and CYP2C9 activity of non-frozen and freeze/thaw HepG2 cells, Fig. 4B, confirming that the freeze/thaw process does not adversely affect the function of these important drug-metabolizing enzymes.

**Fig. 5:**
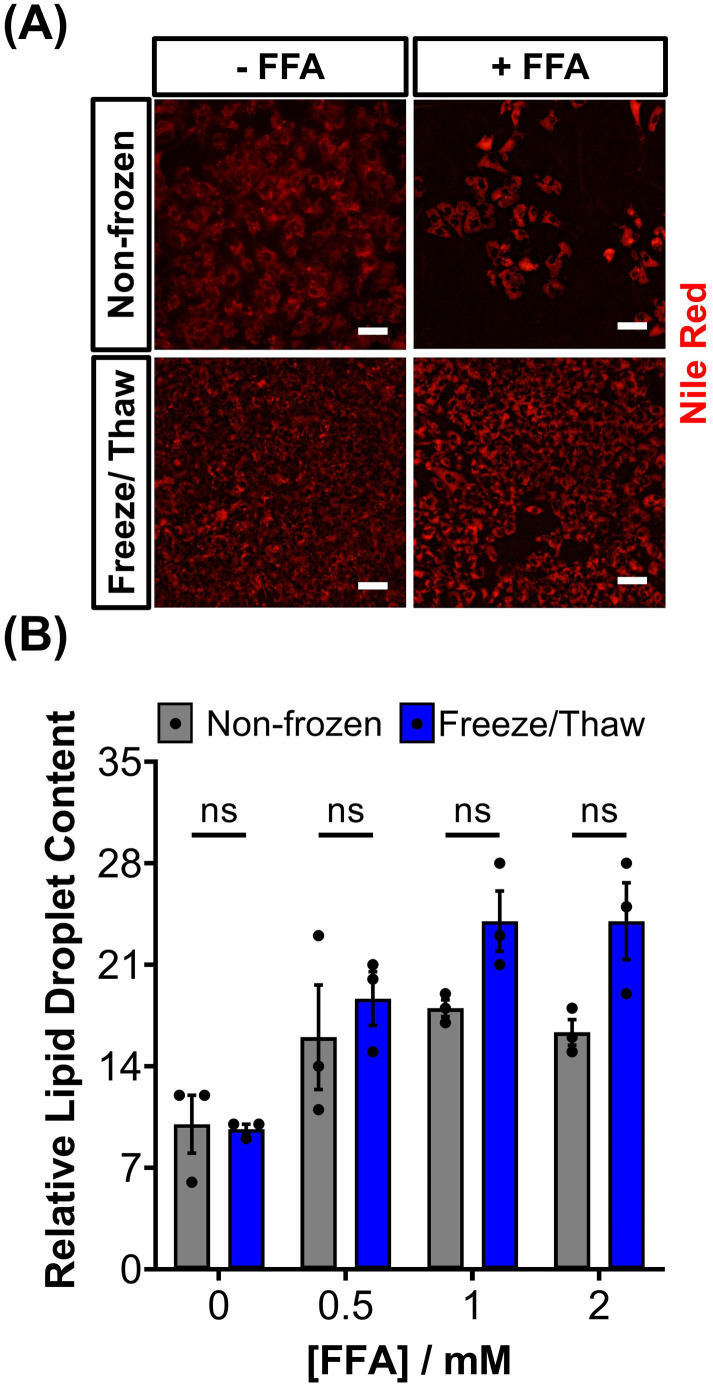
Lipid droplet formation in HepG2 cells after freeze/thaw. (A) Sample fluorescence images of non-frozen and freeze/thaw HepG2 cells treated with (+ FFA) and without (− FFA) a free fatty acid solution (0.5–2 μM sodium palmitate and sodium oleate) and stained with Nile red (scale bar = 100 μm). (B) The relative lipid droplet content of non-frozen (grey) and freeze/thaw (blue) HepG2 cells treated with 0–2 μM FFA was determined by fluorescence measurements recorded from Nile red stained cells. Three biological repeats were completed (ANOVA, Tukey PostHoc ns: *p* ≥ 0.05).

HepG2 cells can accumulate triglycerides in cytosolic lipid droplets, which serves as a proposed marker for steatosis and lipotoxicity upon exposure to free fatty acid (FFA) solutions or drugs.^67^ To assess the impact of the freeze/thaw process on lipid droplet formation, non-frozen and freeze/thaw HepG2 cells were treated with an FFA solution (sodium palmitate and sodium oleate) and stained with Nile red, as shown in Fig. 5A. Both non-frozen and freeze/thaw HepG2 cells exhibited an increase in Nile red fluorescence intensity, indicating enhanced lipid droplet accumulation in response to the FFA solution. The relative lipid content of the cells was quantified and compared, Fig. 5B, revealing no significant differences between the non-frozen and freeze/thaw groups. Images of Nile red-stained HepG2 cells exposed to various concentrations of the FFA solution can be found in the ESI.† FFA concentrations above 0.5 mM were highly cytotoxicity. Therefore, HepG2 cells cryopreserved with an IN maintain their ability to respond normally to FFA solutions, establishing them as a reliable model for studying steatosis and investigating mixtures and drugs that may lead to lipotoxicity.^67^

**Fig. 6:**
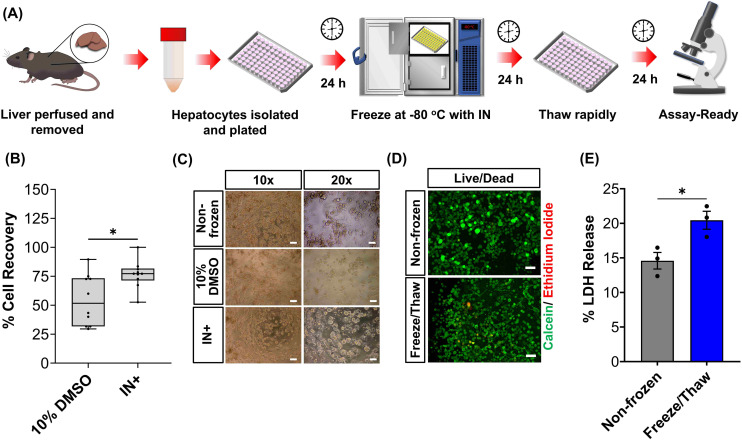
Cryopreservation of primary mouse hepatocytes. (A) Workflow for the cryopreservation of plated mouse hepatocytes adhered to 96 well plates. (B) Percentage cell recovery of mouse hepatocytes using either 10% DMSO or 10% DMSO plus an ice nucleator (IN) ± upper/lower quartiles and min/max of 3 biological repeats and 2 technical repeats (Welch *T*-test; *p ≤ 0.05). (C) Phase contrast images of non-frozen hepatocytes and hepatocyte cryopreserved with 10% DMSO alone or 10% DMSO plus IN (scale bar: ×10 = 100 μm, ×20 = 50 μm). (D) Sample fluorescence images of non-frozen and freeze/thaw (with IN) hepatocytes stained with calcein (green, membrane intact) and ethidium iodide (red, membrane damaged) 24 h post-thaw (scale bar = 100 μm). (E) The lactate dehydrogenase (LDH) released (solid line, maximum) from non-frozen and freeze/thaw (with an IN) hepatocytes into cell culture medium 24 h post-thaw ± SEM of 3 biological repeats (unpaired *T*-test; *p ≤ 0.05).

Human liver cell lines, such as HepG2, are commonly employed in drug metabolism and toxicity studies due to their (essentially) unlimited availability, low cost and extensive knowledge base. However, the limited response of HepG2 gene expression to inducers and low basal CYP450 activity (Fig. 4B and S19†) renders this cell line inadequate as a replacement for primary hepatocytes in drug metabolism studies.^68^ Primary hepatocytes are difficult to isolate, (relatively) expensive and possess limited lifespan so would benefit from cryopreservation to provide readily usable, bankable hepatocytes. Here, primary hepatocytes were isolated from mice livers, plated and cryopreserved with an IN, as illustrated in Fig. 6A. A series of viability studies were carried out 24 hours post-thaw. Approximately 75% of viable hepatocytes (counted using Trypan Blue) were recovered following cryopreservation with 10% DMSO and an IN, a significant increase compared to only ∼50% with DMSO alone, Fig. 6B. Most studies on suspension cryopreservation of mouse hepatocytes solely report viability, rather than cell recovery, which fails to consider the number of cells recovered. However, Lloyd *et al.* achieved a maximum recovery of 54% using DMEM supplemented with 20% FBS and 10% DMSO.^69^ Post-thaw low plating efficiency is also problematic in suspension cryopreserved hepatocytes, ranging from 22–67.5% (compared to 68–82% for fresh hepatocytes) and can decrease over time, due to down-regulation of cell adhesion genes and proteins including β1-integrin and E-cadherin.^70–74^ Consequently, suspension cryopreservation of hepatocytes suffers from the cumulative loss of cells from reduced cell recovery/viability and plating efficiency, whereas cryopreserving hepatocytes as monolayers, using an IN, minimises cell loss by eliminating attachment issues and improving overall recovery.

Phase contrast images in Fig. 6C revealed that freeze/thaw cells maintained their characteristic cuboidal adherent morphology, with granular cytoplasm containing vesicular inclusions and one or more nuclei. Fewer cells were observed in the samples cryopreserved with DMSO alone. Suspension cryopreserved hepatocytes, purchased from a commercial supplier, exhibited a more spherical morphology indicative of reduced attachment capabilities (images can be found in the ESI†), which is typically only observed within a few hours of hepatocyte culture and could relate to the loss of cell adhesion function.^75^ Calcein/ethidium iodide staining and LDH release (into cell culture medium) measurements were completed on freeze/thaw hepatocyte monolayers to probe cryopreservation-induced membrane damage, Fig. 6D and E. An increase in ethidium iodide positive cells and LDH release was observed confirming loss of membrane integrity, although this was kept to less than 5% (1.3-fold increase). In contrast, suspension cryopreserved mouse hepatocytes using University of Wisconsin (UW) solution plus insulin, dexamethasone, 20% FBS and 10% DMSO saw LDH secretion increase by 4-fold, which decreased to 3-fold with the addition of 2 g L^−1^ of glucose.^76^ Moreover, hepatocytes from other species (Human, porcine and rat) cryopreserved in suspension saw increased in LDH release ranging from 1.3–3.5-fold.^69,71,74^ Thus, despite the increased susceptibility of cell monolayers to cryopreservation-induced damage caused by intracellular ice propagation, the membrane integrity of freeze/thaw cells was better preserved using an IN, surpassing the levels observed with most conventional suspension cryopreservation methods reported in the literature.

Hepatocyte cryopreservation has primarily focused on suspension freezing using medium (such as University of Wisconsin, Williams E medium, or Leibowitz L15 medium) supplemented with 10–40% FBS and 10–20% DMSO.^58,71,74,77–79^ Reports on post-thaw function vary significantly, with urea secretion, phase I and II metabolism, albumin/protein synthesis, and ATP content ranging from unchanged to 14% of the values expected from fresh hepatocytes. Therefore, in this study, mouse hepatocyte monolayers cryopreserved with 10% DMSO and IN were evaluated for critical hepatic function 24 hours post-thaw and compared to non-frozen hepatocytes. Additionally, commercially available suspension cryopreserved hepatocytes were included as a benchmark for adequate cellular function in cryopreserved cells.

**Fig. 7:**
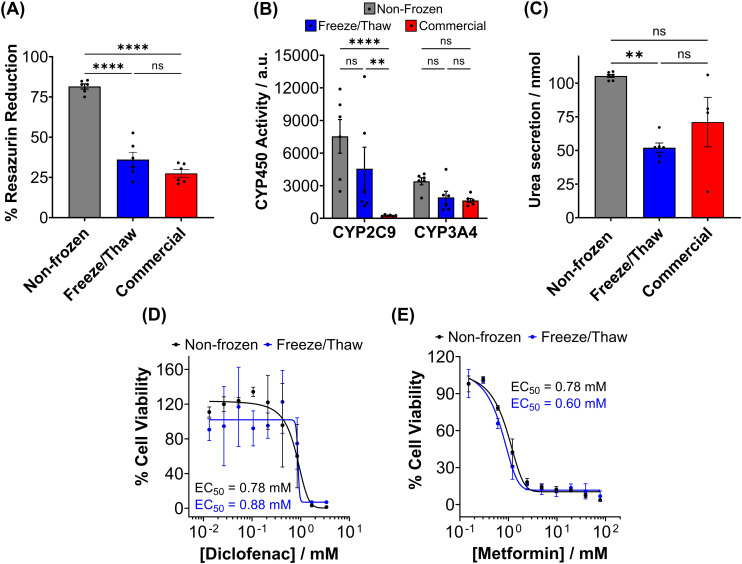
Post-thaw primary hepatocyte function and drug response. Non-frozen (grey) hepatocytes, hepatocytes cryopreserved with 10% DMSO + IN adhered to 96-well plates (blue) and commercially supplied suspension cryopreserved hepatocytes (red) were assessed for (B) resazurin reduction (C) CYP450 activity and (D) urea secretion 24 hours post-thaw. Data is presented as an average ± SEM of 3 biological and 2 technical repeats. Drug–dose response curves were produced for non-frozen (black) and freeze/thaw adherent (blue) hepatocytes treated with (E) diclofenac (0–3.4 μM) and (F) metformin (0–77.4 mM) for 24 hours. Percentage cell viability was calculated using a resazurin reduction assay and reported ± SEM of 3 biological repeats. (ANOVA, Tukey PostHoc; ns: *p* ≥ 0.05, * *p* ≤ 0.05, **p ≤ 0.01, ****p ≤ 0.0001).

The metabolic activity of adherent cryopreserved hepatocytes was assessed using a resazurin reduction assay (Fig. 7A), which measures aerobic respiration. A 56% decrease in metabolic activity was observed compared to non-frozen hepatocytes. Furthermore, the ATP content of cryopreserved hepatocyte monolayers decreased by 70% compared to non-frozen cells (Fig. S20†). Cryopreservation of hepatocytes has been shown to deplete ATP, reduce glycogen storage, and decrease the oxygen consumption rate.^48,57,58^ Although the exact mechanism of ATP reduction is unknown, IIF and osmotic stress has been reported to damage and alter mitochondrial complex 1 activity, which is vital for oxidative phosphorylation (ATP production) and its impairment can lead to ROS generation, decrease in mitochondrial potential and cytochrome C release (which can initiate caspase cascade pathway in apoptosis).^48,80^ De Sousa *et al.* revealed that immediately post-thaw hepatocyte ATP levels are similar to fresh hepatocytes but ATP production is slower over 24 hours, supporting damage to complex 1.^77^ Despite the significant decrease in ATP content and metabolic activity observed in freeze/thaw hepatocyte monolayers, it was still higher than that of commercially cryopreserved hepatocytes.

A ∼50% reduction in phase I metabolism (CYP450 activity) and urea secretion was observed in freeze/thaw cells, Fig. 7B and C. Approximately 25% of the decreases in aerobic respiration, ATP content and differentiated hepatic functions can be attributed to cell loss during the freeze/thaw process. Although cryopreservation is known to reduce urea secretion (25–60% drop) and phase I and II metabolism in hepatocyte, the exact reasons are unknown.^37,44,73,74,81,82^ De Loecker *et al.* reported that post-thaw glycogen basal levels are reduced by 30% 24 h post-thaw and 47% 48 h post-thaw due to an increase in energy demand for cellular repair,^45,48^ which increases viability/membrane integrity but does not always translated to increased hepatic function.^37,44,78^ Thus, increasing post-thaw recovery time above 24 h would likely provide minimal benefits. Despite this, the freeze/thaw adherent hepatocytes exhibited comparable performance to the commercially suspension cryopreserved hepatocytes and are therefore suitable for metabolism studies. To further evaluate the functionality of freeze/thaw primary hepatocytes, a drug toxicity assessment was conducted using diclofenac (Fig. 7E) and metformin (Fig. 7F) as test compounds. The dose–response curves and EC_50_ values obtained from cryopreserved hepatocytes were similar to those of fresh primary hepatocytes, indicating their preserved ability to respond to drug challenges. These findings demonstrate that cryopreserved hepatocytes maintain sufficient functional activity for drug metabolism studies and exhibit comparable performance to commercially available suspension cryopreserved hepatocytes.

## Conclusion

In conclusion, our study demonstrates the successful cryopreservation of HepG2 cells and primary mouse hepatocytes as monolayers in 96 well plates using soluble ice nucleators. This method enables the cells to be “assay ready” directly from the −80 °C freezer with minimal handling, facilitating high-throughput screening and reducing the time burden associated with routine cell culture. Near total cell recovery for HepG2 cells was achieved and approximately 75% recovery for primary hepatocytes, with minimal well-to-well variation, compared to only 29% and ∼50% with DMSO alone, respectively. Post-thaw HepG2 cells retained normal proliferative capacity and differentiated hepatic functions, making them suitable for toxicological screening. Furthermore, non-frozen and freeze/thaw HepG2 cells exhibited near-identical EC_50_ values against a panel of pharmaceutically relevant drugs, indicating that the cryopreservation process did not affect their drug response. Although post-thaw primary hepatocytes experienced reduced metabolic activity, CYP450 activity and urea secretion, their performance was comparable to suspension cryopreserved hepatocytes provided by cell manufacturers. This work highlights the effectiveness of addressing a key biophysical mode of damage during micro-well plate cryopreservation, namely super cooling, to allow cell banking and distribution in a ready-to-use format. Our method provides an efficient and sustainable cryopreservation method for hepatocyte-derived cell lines and primary hepatocytes, with broad applications in the drug discovery process. By reducing the time and resources required for thawing, propagation, and plating of cryopreserved cells, while ensuring phenotypically identical cells for consistent and reliable screening results, this cryopreservation method offers significant advantages. The implementation of this method in high-throughput screening using 96 well plates allows for simultaneous analysis of multiple test compounds, increasing the efficiency and productivity of drug screening assays. Further optimization and validation of this cryopreservation technique may open up new possibilities for other cell types and expand its application in various fields of cell-based research and testing.

## Experimental section

For complete experimental methods see the ESI.†

### Materials

Minimum Essential Medium Eagle with Earle's salts, l-glutamine and sodium bicarbonate (M4655); fetal bovine serum, non-USA origin (F7524); MEM non-essential amino acid solution (100×) (M7145); Type I solution from rat tail (C3867); DMSO hybri-max™ (D2650); phenol-free DMEM/F-12 medium (1121041-025); carbamazepine (1093001); urea assay kit (MAK006); sodium palmitate (P9767); sodium oleate (07501); bovine serum albumin (A3294); Dulbecco's phosphate buffered saline, modified (DPBS), w/o calcium chloride and magnesium chloride (D8537); acetaminophen (A5000); valproic acid (V0033000); doxorubicin hydrochloride (D2975000); metformin hydrochloride (M0605000); phenformin hydrochloride (1003386493); Nile red (102011796); diclofenac sodium (287840-1GM); Corning XT CoolSink 96F (CLS432070); cholesterol (C3045); Liberase™ (5401119001) were purchased from Merck, Gillingham, UK. Amphotericin B, penicillin, streptomycin (PSA) (11570486); trypsin (0.25%) and EDTA (1 mM) (25200 072); rifampicin (BP2679); HEPES buffer solution (11560496) were purchased from Fisher Scientific, Loughborough, UK. CyQUANT™ LDH Cytotoxicity Assay kit (C20300); LIVE/DEAD™ Viability/Cytotoxicity Kit, for mammalian cells (L3224); CellEvent caspase-3/7 detection reagent (C10723); HBSS with no Ca^2+^, no Mg^2+^ and no phenol red (14175095); HBSS with Ca^2+^ and Mg^2+^ and no phenol red (14025092); William's E medium, no phenol red (A1217601); Primary Hepatocyte Thawing and Plating Supplements (CM3000); Primary Hepatocyte Maintenance Supplements (CM4000) were purchased from Thermo Fisher Scientific, Loughborough, UK. Trypan blue solution 0.4% (25-900-C1); trehalose dihydrate (T9531) and resazurin tablets (CHE3158) were purchased from Scientific Laboratory Supplies, Nottingham, UK. P450-Glo™ CYP2C9 Assay (V8792) and P450-Glo™ CYP3A4 Assay (V9002) were purchased from Promega, Wisconsin, USA. Sambucus nigra (“Elder”) pollen and Carpinus.

### Cell line culture

Human liver hepatocellular carcinoma cells (HepG2, ECACC 85011430) were cultured in Eagle's minimum essential medium (EMEM) supplemented with 10% foetal bovine serum (FBS), 1% MEM non-essential amino acids (NEAA), 100 units per mL penicillin, 100 μg mL^−1^ streptomycin, and 250 ng mL^−1^ amphotericin B (1% PSA). Cells were incubated at 37 °C and 5% CO_2_ and passaged every 3–4 days, before reaching 70–80% confluency. Cells were dissociated using a balanced salt solution containing trypsin (0.25%) and EDTA (1 mM). Mycoplasma contamination was tested routinely with a MycoAlert Mycoplasma Detection Kit 150 (Lonza, Basel, Switzerland). HepG2 cells were cultured on clear 96-well plates coated with rat tail type I collagen (100 μg mL^−1^) at varying densities outlined throughout.

### Statistical analysis

Statistical analysis and data visualization were performed using GraphPad Prism 8 (La Jolla, California, USA). The Shapiro-Wilk test and Levene test were utilized to assess the normality of the data and equality of variances between groups. If the data met the assumptions of normality and equal variance, it was reported as mean ± SEM. Statistical significance between the means of 2 groups were assessed using an unpaired (independent) *T*-test and for 2+ groups a one-way analysis of variance (ANOVA), followed by Tukey's *post hoc* analysis, was used. In cases where the assumption of equal variances was violated but data still exhibited normal distribution, box plots were generated, representing the median, upper and lower quartiles, and minimum and maximum limits. Statistical significance between means was determined using Welch's *t*-test. The significance levels were represented as follows: not significant (ns) for *p* ≥ 0.05, * for *p* ≤ 0.05, ** for *p* ≤ 0.01, and **** for *p* ≤ 0.0001. Dose–response curves were generated using nonlinear regression with a variable slope (four parameters) fitted by the least squares regression method, without any weighting. GraphPad determined the EC_50_ values based on the fitting process.

### Primary mouse hepatocyte isolation and culture

Mice were bred at the University of Warwick after local AWERB and Home Office approvals (PP3644080). Hepatocytes were isolated from adult (8- to 12-weeks old) male and female C57BL/6NCrl mice *via* a two-step perfusion process. Perfusion was carried out through the vena cava at a flow rate of 6 mL min^−1^ for 10 min with buffers warmed to 42 °C. Perfusion buffer I consisted of HBSS with no Ca^2+^ or Mg^2+^ supplemented with EDTA (0.5 mM) and HEPES (25 mM). Perfusion Buffer II (digestion buffer) consisted of HBSS with Ca^2+^ or Mg^2+^ supplemented with HEPES (25 mM) and Liberase™ (40 μg mL^−1^). The liver was removed and dissociated in cold suspension buffer (digestion buffer without Liberase™). The cells were passed through a 70 μm filter and centrifuged at 50*g* for 2 min at 4 °C. After two washes with plating medium (Williams E supplemented with CM3000 thawing/plating supplement pack), cells were plated in 96 well plate coated with rat tail type I collagen (100 μg mL^−1^) at a density of 30k cell per well for 4 hours. The medium was exchanged for maintenance medium (Williams E medium supplemented with CM4000 maintenance pack) and the cells were incubated for a further 24 hours. Gibco™ Mouse (CD-1) Cryopreserved Hepatocytes, Plateable Male (6–12 weeks) (10890041) were purchased from Fisher Scientific (Loughborough, UK) to compare our cryopreservation approach to commercial suspension cryopreserved hepatocytes. Cells were thawed with thawing medium and plated at a density of 30k cell per well in 96-well plates. The medium was replaced with maintenance medium after 4 hours and incubated for a further 24 hours.

### Cryopreserving and thawing cell monolayers

HepG2 cells (density dependent on assay) and mouse hepatocytes (30k cell per well) were plated on rat tail type I collagen (100 μg mL^−1^) coated 96 well plates and incubated for 24 hours. The cryoprotectant solution was prepared in advance. Hornbeam pollen (0.8 g) was suspended in sterile water (10 mL) for 24 h at 4 °C. The solution was sterile filtered and mixed 1 : 1 with either Eagle's minimum essential base medium (HepG2) or Williams E base medium (Mouse hepatocytes) supplemented with 20% FBS and 20% DMSO (final concentration 10%) to produce the cryopreservation formulation. The cell culture medium of the cells was replaced with 100 μL of the prechilled cryopreservation formulation (4 °C) and the plates were placed on a Corning XT CoolSink 96F and in a −80 °C freezer overnight. HepG2 cells and hepatocytes were also cryopreserved with 10% DMSO alone in cell culture medium for comparison. To thaw the cells, 150 μL of warm complete/thawing cell culture medium (37 °C) was added and the plates were placed in the incubator for 12 min. The solutions were replaced with 200 μL of warm complete (HepG2)/maintenance (Hepatocyte) medium and the cells were placed in the incubator for a further 24 h. Phase contrast images were obtained before and after cryopreservation.

### Percentage cell recovery/growth curves

HepG2 cells were cryopreserved between 1.6–30k cell per well and hepatocytes at a density of 30k cell per well. Cell counts were performed immediately before cryopreservation and 24 hours post-thaw to calculate percentage cell recovery. To perform cell counts, cell dissociation was completed using trypsin (0.25%) and EDTA (1 mM) and viable cells were stained with Trypan Blue (0.2% for HepG2 and 0.04% for hepatocytes). Freeze/Thaw and non-frozen HepG2 cells were counted daily until 80–90% confluency was reached to determine proliferation rates.

### Live/Dead™ staining

Non-frozen and Freeze/Thaw HepG2 cells (15k cell per well) and mouse hepatocytes (30k cell per well) were washed twice with DPBS and stained with ethidium iodide (2 μM) and calcein (2 μM) at RT for 40 min. HepG2 cells treated with ice cold 70% methanol for 30 min was also stained with both dyes as a positive control for comparison. Cells were imaged on an Olympus CX41 microscope equipped with a UIS-2 10×/0.45/∞/0–2/FN22 lens using a phase contrast channel and with blue (calcein) and green (ethidium) excitation lasers. Cells were counted using ImageJ. Values were reported as percentage live cells relative to the total number of cells.

### Lactate dehydrogenase release

Non-frozen and Freeze/Thaw HepG2 cells (0–15k cell per well) and mouse hepatocytes (30k cell per well) were tested for enzyme leakage using the CyQUANT™ LDH Cytotoxicity Assay kit (ThermoFisher Scientific). Either 10 μL ultrapure water, to determine the release of LDH to the cell culture medium (‘Spontaneous LDH Release’), or 10 μL of 10× Lysis Buffer, provided by the CyQUANT™ kit, to determine the total LDH content of the cells (‘Maximum LDH Release’), was added to wells. The cells were incubated at 37 °C for 45 min and 50 μL of each sample medium (Spontaneous and Maximum LDH Activity Controls) was added to a new 96-well plate in triplicate (three technical repeats). The Reaction Mixture provided by the CyQUANT™ LDH Cytotoxicity Assay kit was added to each well and mixed by tapping. The plates were incubated at RT for 30 min and 50 μL of stop solution was added. Absorbance measurements were recorded on a BioTek Synergy HT microplate reader at 490 nm and 680 nm (background measurement). Both the Spontaneous and Maximum LDH Release Control Absorbance values were plotted following subtraction of the background absorbance measurement. Non-frozen control cell samples were also analysed for comparison.

### Caspase-3/-7 activation

Freeze/thaw HepG2 cells were incubated with CellEvent Caspase-3/7 Detection Reagent (5 μM) was added to HepG2 cells immediately after thawing and imaged 2, 4, 8 and 24 h post-thaw using the phase contrast channel and blue excitation laser of an Olympus CX41 microscope. Non-frozen HepG2 cells treated with and without staurosporine (2 μM, 24 h) were also imaged to provide a positive and negative control, respectively. Cells were counted using ImageJ and values were reported as percentage caspase positive cells relative to the total number of cells.

### Resazurin reduction metabolic assay

Freeze/thaw HepG2 cells (0–15k cell per well) were incubated with resazurin solution (100 μL) for 4 h and freeze/thaw mouse hepatocytes (30k cell per well) and Gibco™ Mouse (CD-1) Cryopreserved Hepatocytes (30k cell per well) for 24 h. Resazurin solution was prepared by dissolving 1 resazurin tablet (Scientific Laboratory Supplies) in 50 mL of phenol-free DMEM/F-12 medium supplemented with 10% FBS and sterile filtered. Absorbance measurements were obtained at 570 nm and 600 nm using a BioTek Synergy HT microplate reader. Non-frozen cells were also measured for comparison and resazurin solution alone for background subtraction. See ESI† for resazurin reduction calculations. Resazurin reduction was calculated using the following equation:
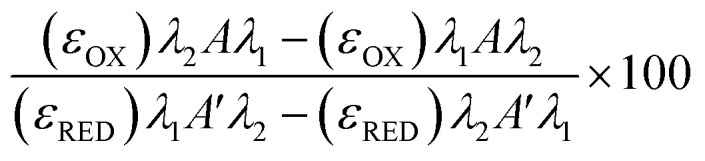
*λ*_1_ = 570 nm, *λ*_2_ = 600 nm; *Aλ*_1_ & *Aλ*_2_ = Absorbance of test sample, control or media alone at 570 nm and 600 nm; *A*′*λ*_1_ & *A*′*λ*_2_ = Absorbance of media alone at 570 nm and 600 nm; (*ε*_OX_) & (*ε*_RED_) = Molar extinction coefficient of resazurin at respective wavelengths.

### Drug dose–response

Non-frozen and freeze/thaw HepG2 cells (15k cell per well) were treated with acetaminophen (0–92.6 mM), diclofenac (0–3.4 mM), doxorubicin (0–9.2 mM), metformin (0–310 mM), phenformin (0–413 mM) and valproic acid (0–50 mM) and placed in a humidified environment at 37 °C and 5% CO_2_ for 24 h. Non-frozen and freeze/thaw hepatocytes were treated with diclofenac (0–3.4 μM) and metformin (0–77.4 mM) and placed in a humidified environment at 37 °C and 5% CO_2_ for 24 h. The cell culture medium was replaced with 100 μL of resazurin solution and the cells were incubated for 2 h in a humidified environment at 37 °C and 5% CO_2_. Fluorescence measurements were recorded with excitation and emission at 530/25 nm and 590/30 using a BioTek Synergy HT microplate reader. Wells with resazurin solution alone were also measured for background subtraction and cells untreated with pharmaceutically active compounds were measured as a maximum resazurin reduction value. Percentage cell viability was reported using the fluorescence readings using the following equation:
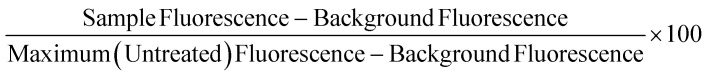


### Urea secretion

Urea measurements for non-frozen and freeze/thaw HepG2 cells were taken after 1, 4 and 7 days of culture using the Sigma-Aldrich Urea Assay kit (MAK006). Urea measurements for non-frozen and freeze/thaw hepatocytes and Gibco™ Mouse (CD-1) Cryopreserved Hepatocytes were recorded after 1 day of culture. Briefly, the supernatant collected (2 μL) was diluted in urea assay buffer (48 μL) and either complete reaction mix was added (50 μL: urea assay buffer, 42 μL; peroxidase substrate, 2 μL; enzyme mix, 2 μL; developer, 2 μL; converting enzyme, 2 μL) or reaction mix without converting enzyme was added (50 μL), to subtract backgrounds generated by ammonium ion, NAD+/NADP+, and pyruvate. The plate was mixed and incubated for 60 min in a humidified environment at 37 °C and 5% CO_2_. Absorbance measurements were recorded at 570 nm using a BioTek Synergy HT microplate reader. Values were compared against a urea calibration curve of 0–5 nmol per well and reported as nmol per μL and nmol per μL per 10^6^ cells.

### Lipid droplet staining

Non-frozen and freeze/thaw HepG2 cells (15k cell per well) were incubated in a humidified environment at 37 °C and 5% CO_2_ with complete cell culture medium supplemented with a free fatty acid solution (100 μL) consisting of 0–2 μM sodium palmitate and sodium oleate (1 : 1) and 1% bovine serum albumin (BSA) for 24 h. The cells were washed with DPBS (×3) and stained with Nile red (100 μL, 15 μM) diluted in phenol-free DMEM base medium for 30 min at RT. Cells were washed with DPBS (×3) and imaged on an Olympus CX41 microscope equipped with a UIS-2 20×/0.45/∞/0–2/FN22 lens using a phase contrast channel and green excitation laser. The DPBS was removed, and cells were allowed to dry for 15 min at RT. Fluorescence measurements were recorded using a 530/25 nm excitation laser and 590/35 nm emission filter using a BioTek Synergy HT microplate reader. Fluorescence of Nile red is directly proportional to the number of lipid droplets.

### Cytochrome P450

Non-frozen and freeze/thaw HepG2 cells (15k cell per well), non-frozen and freeze/thaw hepatocytes (30k cell per well) and Gibco™ Mouse (CD-1) Cryopreserved Hepatocytes were assessed for CYP3A4 and CYP2C9 activity. To determine CYP450 activity, CYP3A4 and CYP2C9 were measured using the corresponding Promega CYP450 kits (V8792 and V9002). Briefly, the medium was replaced with a culture medium containing a luminogenic CYP substrate, either CYP3A4/Luciferin-IPA (3 μM, 50 μL, 1 h) or CYP2C9/Luciferin-H (100 μM, 50 μL, 3 h). The CYP substrate was also added to empty wells as a background measurement. The culture medium containing the CYP substrate (25 μL) was transferred to an opaque white 96 well plate, and luciferin detection reagent (25 μL) was added for 20 min at RT. Luminescence was measured on a TECAN Spark microplate reader with 1 s integration time.

## Conflicts of interest

MIG and TC are directors and shareholders of Cryologyx Ltd which funded aspects of this work. MIG is a named inventor on a patent application relating to this work.

## Supplementary Material

BM-011-D3BM01046E-s001
