# Supplementary material for: Cryopreservation of assay-ready hepatocyte monolayers by chemically-induced ice nucleation: preservation of hepatic function and hepatotoxicity screening capabilities
Source: Biomater Sci. 2023 Oct 16;11(23):7639–54. doi: 10.1039/d3bm01046e (PMC10661096; doi:10.1039/d3bm01046e)
Supplement: BM-011-D3BM01046E-s001 [file BM-011-D3BM01046E-s001.pdf]

Electronic Supplementary Material (ESI) for Biomaterials Science.
This journal is © The Royal Society of Chemistry 2023

Electronic Supporting Information

# Cryopreservation of Assay-Ready Hepatocyte Monolayers by Chemically-Induced Ice Nucleation: Preservation of Hepatic Function and Hepatotoxicity Screening Capabilities

Ruben M. F. Tomás,[a] Robert Dallman,[a] Thomas R. Congdon,[c] and Matthew I. Gibson[a,b*]

[a] *Division of Biomedical Sciences, Warwick Medical School, University of Warwick, Gibbet Hill Road, Coventry, CV4 7AL, UK;*

[b] *Department of Chemistry, University of Warwick, Gibbet Hill Road, Coventry, CV4 7AL, UK;*

[c] *Cryologyx Ltd, 71-75 Shelton Street, London, UK, WC2H 9JQ, UK*

*Corresponding Author m.i.gibson@warwick.ac.uk

# 1 Experimental Section

## 1.1 Materials

Minimum Essential Medium Eagle with Earle′s salts, L-glutamine and sodium bicarbonate (M4655); Fetal Bovine Serum, non-USA origin (F7524); MEM Non-essential Amino Acid Solution (100x) (M7145); Type I solution from rat tail (C3867); DMSO hybri-max$^{TM}$ (D2650); phenol-free DMEM/F-12 medium (1121041-025); Carbamazepine (1093001); Urea Assay kit (MAK006); sodium palmitate (P9767); sodium oleate (07501); Bovine Serum Albumin (A3294); Dulbecco's Phosphate Buffered Saline, Modified (DPBS), w/o calcium chloride and magnesium chloride (D8537); Acetaminophen (A5000); valproic acid (V0033000); doxorubicin hydrochloride (D2975000); metformin hydrochloride (M0605000); Phenformin hydrochloride (1003386493); Nile red (102011796); Diclofenac Sodium (287840-1GM); Corning XT CoolSink 96F (CLS432070); Cholesterol (C3045); Liberase$^{TM}$ (5401119001) were purchased from Merck, Gillingham, UK. Amphotericin B, Penicillin, Streptomycin (PSA) (11570486); trypsin (0.25%) and EDTA (1 mM) (25200 072); Rifampicin (BP2679); HEPES buffer solution (11560496) were purchased from Fisher Scientific, Loughborough, UK. CyQUANT™ LDH Cytotoxicity Assay kit (C20300); LIVE/DEAD™ Viability/Cytotoxicity Kit, for mammalian cells (L3224); CellEvent Caspase-3/7 Detection Reagent (C10723); HBSS with no $Ca^{2+}$, no $Mg^{2+}$ and no phenol red (14175095); HBSS with $Ca^{2+}$ and $Mg^{2+}$ and no phenol red (14025092); William's E Medium, no phenol red (A1217601); Primary Hepatocyte Thawing and Plating Supplements (CM3000); Primary Hepatocyte Maintenance Supplements (CM4000) were purchased from Thermo Fisher Scientific, Loughborough, UK. Trypan blue solution 0.4% (25-900-C1), Trehalose dihydrate (T9531) and resazurin tablets (CHE3158) were purchased from Scientific Laboratory Supplies, Nottingham,

UK. P450-Glo™ CYP2C9 Assay (V8792) and P450-Glo™ CYP3A4 Assay (V9002) were purchased from Promega, Wisconsin, USA. Sambucus nigra ("Elder") pollen and Carpinus betulus ("Hornbeam") pollen were purchased from Pharmallegra, Lišov, Czech Republic.

## 1.2 Cell Line Culture

Human liver hepatocellular carcinoma cells (HepG2, ECACC 85011430) were cultured in Eagle's minimum essential medium (MEM) supplemented with 10% foetal bovine serum (FBS), 1% MEM non-essential amino acids (NEAA), and 1% Amphotericin B, Penicillin, Streptomycin (PSA). Human Caucasian lung carcinoma cells (A549, ATCC) were cultured in Ham's F-12K (Kaighn's) medium (F-12K) supplemented with 10% FBS and 100 units.mL$^{-1}$ penicillin, 100 μg·mL$^{-1}$ streptomycin, and 250 ng·mL$^{-1}$ amphotericin B (1% PSA). Cells were incubated at 37 °C and 5% $CO_2$ and passaged every 3–4 days, before reaching 70–80% confluency. Cells were dissociated using a balanced salt solution containing trypsin (0.25%) and EDTA (1 mM). Mycoplasma contamination was tested routinely with a MycoAlert Mycoplasma Detection Kit 150 (Lonza, Basel, Switzerland).

## 1.3 Screening Conditions for Cryopreserving Hepatocyte Monolayers

Confluent HepG2 monolayers were produced by seeding 30k cells/well in 96 well plates coated with 12.7 μg/cm$^2$ Type I collagen from rat tail. Cells were incubated for 24 h to allow attachment to the substrate. HepG2 monolayers were subsequently cryopreserved with various cryoprotectants to select the ideal cryopreservation formation: (**1.3.1**) 10% DMSO alone or 10% DMSO and polyampholyte; (**1.3.2**) 10% DMSO and cholesterol monohydrate; (**1.3.3**) 10% DMSO and hornbeam pollen washing water.

### 1.3.1 Polyampholyte

A previous method developed by Gibson *et al.* was attempted due to its success in the cryopreservation of multiple cell lines in 24-well plate format.[1] Cell culture medium was removed from the HepG2 cells and replaced with 200 μL of MEM base medium supplemented with 10% FBS, 10% dimethyl sulfoxide (DMSO) and 0–200 mg·mL$^{-1}$ polyampholyte. The cells were incubated at RT for 10 mins and the cryoprotectant solution was removed, leaving a residual thin film of cryoprotectant. The 96 well plates were positioned on a Corning XT CoolSink 96F and placed in a −80 °C freezer overnight. As a control, HepG2 monolayers were cryopreserved following the same protocol with 10% DMSO alone, the current 'gold' standard cryoprotectant. Following 24 h, cells were removed from the −80 °C, immediately thawed with 200 μL of warm complete cell media (37 °C) and placed in the incubator for 24 h. Percentage cell recovery was subsequently determined by dissociating the cells with trypsin (0.25%) and EDTA (1 mM), counting membrane intact cells stained with trypan blue (0.2%) and comparing cell counts recorded immediately before and 24 hours post freeze/thaw. Two biological and four technical repeats were completed.

### 1.3.2 Cholesterol Monohydrate

The cell culture medium of HepG2 monolayers was removed and replaced with a cryopreservation formulation (50 μL) consisting of MEM base medium supplemented with 10% FBS, 10% DMSO and 25–25e$^{-7}$ μg·mL$^{-1}$ of cholesterol monohydrate crystals (prepared by re-crystallization of cholesterol in ethanol, as previously described).[2] The 96 well plates were positioned on a Corning XT CoolSink 96F and placed in a −80 °C freezer overnight. Following 24 h, cells were removed from the −80 °C freezer, thawed with 150 μL of warm complete cell media (37 °C) and placed in the incubator for 12 mins. The medium was replaced with 200 μL of complete cell medium and placed in the incubator for 24 h. Percentage cell recovery was determined as described in 1.3.1.

### 1.3.3 Hornbeam Pollen Washing Water

Hornbeam pollen washing water (PWW) was prepared in advance by weighing 0.8 g of hornbeam pollen in $H_2O$. The PPW solutions were kept at 4 °C for 24 h, sterile filtered and mixed 1:1 with MEM base medium supplemented with 20% FBS and 20% DMSO. The chilled cryopreservation solutions were added to the HepG2 monolayers (100 μL) and immediately placed on a Corning XT CoolSink 96F and frozen in a −80 °C freezer for 24 h. Cells were removed from the −80 °C and 150 μL of warm complete cell medium (37 °C) was added. The cells were placed in an incubator for 12 min, to allow thawing, and the solutions were replaced with 200 μL of warm complete cell medium (37 °C). After 24 hours, percentage cell recovery was determined as described in 1.3.1.

## 1.4 Cell Recovery of Optimised Freeze/Thaw HepG2 Monolayers

HepG2 cells were seeded either as confluent monolayers (30k cells/well) or at varying cell densities (dependent on the assays described below) in Type I collagen coated 96 well plates (12.7 μg/cm$^2$). Cells were incubated for 24 h to allow attachment to the substrate. Hornbeam pollen washing water (PWW) was prepared by weighing 0.8 g of hornbeam pollen and suspending it in $H_2O$ for 24 h at 4 °C. The PWW solutions were sterile filtered, to remove particles, and mixed 1:1 with MEM base medium supplemented with 20% FBS and 20% DMSO (final concentration 10%) to produce the cryopreservation formulation. The medium was removed from the HepG2 monolayers and replaced with 100 μL of the prechilled cryopreservation formulation (4 °C). The cells were placed on a Corning XT CoolSink 96F and in a −80 °C freezer overnight. Cells were also cryopreserved with this method in a solution containing MEM base medium supplemented with 10% FBS and 10% DMSO (no pollen) for comparison against the 'gold standard' cryoprotectant alone. Cells were removed from the −80

°C and 150 μL of warm complete cell medium (37 °C) was added. The cells were placed in the incubator for 12 mins, to allow thawing, and the solutions were replaced with 200 μL of warm complete cell medium (37 °C). The cells were placed in the incubator for a further 24 h. Percentage cell recovery was determined by dissociating cells with trypsin (0.25%) and EDTA (1 mM), staining the cells with trypan blue (to count membrane intact cells) and comparing cell counts immediately before and 24 h after freeze/thaw. A549 cells were cryopreserved using the same approach as confluent monolayers (20k cells/well) and varying cell densities (outlined throughout) as another comparative cell line, using Ham's F-12K (Kaighn's) Medium supplemented with 10% FBS and 1% PSA as the complete growth medium and Ham's F-12K (Kaighn's) Medium alone as the base medium. Cell recovery values were also obtained for plates containing A549 and HepG2 cells stored for over 1 month in a -80 °C freezer.

## 1.5 Assessing Cellular Health

### 1.5.1 Growth Curves

HepG2 cells (7.5k cell/well) were seeded in collagen (12.7 μg/cm$^2$) coated 96 well plates and A549 cells (7.5k cell/well) were seeded in collagen uncoated 96 well plates. The plates were freeze/thawed as described in section 1.4. Daily cell counts were performed, using Trypan blue (0.2%), on the cryopreserved cells, and non-frozen cells for comparison, until 80–90% confluency was reached to determine proliferation rates.

### 1.5.2 Resazurin Reduction Metabolic Assay

HepG2 cells (0–15k cell/well) were seeded in collagen (12.7 μg/cm$^2$) coated 96 well plates and A549 cells (0–15k cell/well) were seeded in collagen uncoated 96 well plates. The plates were freeze/thawed as described in section 1.4. The cell culture medium was replaced with 100 μL of resazurin solution, prepared by dissolving 1 resazurin tablet in 50 mL of phenol-free

DMEM/F-12 medium supplemented with 10% FBS and sterile filtered. The cells were incubated for 4 h in a humidified environment at 37 °C and 5% $CO_2$. Absorbance measurements were obtained at 570 nm and 600 nm and fluorescence measurements were recorded with excitation and emission at 530/25 nm and 590/30 using a BioTek Synergy HT microplate reader. Wells with resazurin solution alone were also measured for background subtraction. A resazurin reduction assay of non-frozen cells was also completed for comparison. Results were reported as relative resazurin reduction (absorbance) or normalised resazurin reduction (fluorescence).

Calculating Percentage Resazurin Reduction:

$$\frac{(\varepsilon_{OX})\lambda_2 A\lambda_1 - (\varepsilon_{OX})\lambda_1 A\lambda_2}{(\varepsilon_{RED})\lambda_1 A'\lambda_2 - (\varepsilon_{RED})\lambda_2 A'\lambda_1} \text{x } 100$$

$\lambda_1 = 570\, nm, \lambda_2 = 600\, nm$

$A\lambda_1 \& A\lambda_2 = Absorbance\ of\ test\ sample, control\ or\ media\ alone\ at\ 570\ nm\ and\ 600\ nm$

$A'\lambda_1 \& A'\lambda_2 = Absorbance\ of\ media\ alone\ at\ 570\ nm\ and\ 600\ nm$

$(\varepsilon_{OX})\ \&\ (\varepsilon_{RED}) = Molar\ extinction\ coefficient\ of\ resazurin\ at\ respective\ wavelengths$

1.5.3 Calcein/ Ethidium Iodide Staining

HepG2 cells (15k cell/well) were seeded in collagen coated 96 well plates and A549 cells (15k cell/well) were seeded in collagen uncoated 96 well plates. The plates were freeze/thawed using the optimised protocol described in 1.4. The cell culture medium was replaced with 100 µL of DMEM/F-12 medium supplemented with 10% FBS, ethidium iodide (2 µM) and calcein (2 µM) and incubated at RT for 40 min. Non-frozen HepG2 cells treated with ice cold 70% methanol for 30 mins were also stained with ethidium iodide and calcein as a negative and positive control, respectively. Cells were imaged on an Olympus CX41 microscope equipped with a UIS-2 20x/0.45/∞/0–2/FN22 lens using a phase contrast channel and with blue (calcein)

and green (ethidium) excitation lasers. Cells were counted using ImageJ. Values were reported as percentage live cells relative to the total number of cells.

### 1.5.4 Caspase-3/-7 real-time Activation

HepG2 cells (15k cell/well) were seeded in collagen coated 96 well plates and A549 cells (15k cell/well) were seeded in collagen uncoated 96 well plates. The plates were freeze/thawed using the optimised protocol described in 1.4. However, after the 12 min thawing period the solution was replaced with 100 µL of warm complete cell culture medium (37 °C) additionally supplemented with CellEvent Caspase-3/7 Detection Reagent (5 µM). Images were taken at 2, 4, 8 and 24 h time intervals on an Olympus CX41 microscope using a phase contrast channel and a blue excitation laser. Non-frozen HepG2 cells treated with staurosporine (2 µM, 24 h) were used as a positive control. Cells were counted using ImageJ and values were reported as percentage caspase positive cells relative to the total number of cells.

### 1.5.5 Lactate Dehydrogenase (LDH) Release

HepG2 cells (0–15k cell/well) were seeded in collagen coated plates and freeze/thawed using the optimised protocol in 1.4. After 24 hours, for each cell density tested, either 10 µL of ultrapure water was added, to determine the release of LDH to the cell culture medium (‘Spontaneous LDH Release’), or 10 µL of 10X Lysis Buffer, provided by the CyQUANT™ LDH Cytotoxicity Assay kit (ThermoFisher Scientific,), was added to determine the total LDH content of the cells (‘Maximum LDH Release’). The HepG2 cells were incubated at 37 °C for 45 min and 50 µL of each sample medium (Spontaneous and Maximum LDH Activity Controls) was added to a new 96-well plate in triplicate (three technical repeats). The Reaction Mixture provided by the CyQUANT™ LDH Cytotoxicity Assay kit was added to each well and mixed by tapping. The plates were incubated at RT for 30 min and 50 µL of stop solution was added. Absorbance measurements were recorded on a BioTek Synergy HT microplate

reader at 490 nm and 680 nm (background measurement). Both the Spontaneous and Maximum LDH Release Control Absorbance values were plotted following subtraction of the background absorbance measurement. Non-frozen control cell samples were also analysed following this protocol for comparison.

## 1.6 Preservation of Differentiated Hepatic Function in HepG2 Cells

### 1.6.1 Urea Secretion

HepG2 cells were seeded at 15k cell/well in collagen coated plates and freeze/thawed using the optimised protocol in section 1.4. Urea measurements were taken at day 1, 4 and 7 using the Sigma-Aldrich Urea Assay kit (MAK006). The supernatant of freeze/thaw and non-frozen cells were collected (2 μL) and diluted in Urea assay buffer (48 μL); this was completed four times for each sample. Complete reaction mix was added to two wells (50 μL: Urea assay buffer, 42 μL; Peroxidase substrate, 2 μL; Enzyme mix, 2 μL; Developer, 2 μL; Converting enzyme, 2 μL) and reaction mix without converting enzyme was added to two wells for each sample (50 μL: Urea assay buffer, 44 μL; Peroxidase substrate, 2 μL; Enzyme mix, 2 μL; Developer, 2 μL), to subtract backgrounds generated by ammonium ion, NAD+/NADP+, and pyruvate. The plate was mixed (by pipetting) and incubated for 60 min in a humidified environment at 37 °C and 5% $CO_2$. Absorbance measurements were recorded at 570 nm using a BioTek Synergy HT microplate reader. Values were compared against a urea calibration curve of 0–5 $nmol.well^{-1}$ and reported as $nmol.\mu L^{-1}$ and $nmol.\mu L^{-1}$ per $10^6$ cells.

### 1.6.2 Lipid Droplet Staining

HepG2 cells were seeded at 15k cell/well in collagen coated plates and freeze/thawed using the optimised protocol in section 1.4. The cell medium was replaced with free fatty acid solution (100 μL) consisting of 0–2 μM sodium palmitate and sodium oleate (1:1) in complete MEM

medium further supplemented with 1% bovine serum albumin (BSA) and the cells were placed in a humidified environment at 37 °C and 5% $CO_2$ for 24 h. The cells were washed with DPBS (x3) and stained with 100 µL of 15 µM Nile red (prepared from a 30 mM stock in ethanol) in phenol-free DMEM base medium for 30 mins at RT. Cells were washed with DPBS (x3) and imaged on an Olympus CX41 microscope equipped with a UIS-2 20x/0.45/∞/0−2/FN22 lens using a phase contrast channel and green excitation laser. The DPBS was removed, and cells were allowed to dry for 15 min at RT. Fluorescence measurements were recorded using a 530/25 nm excitation laser and 590/35 nm emission filter using a BioTek Synergy HT microplate reader. Non-frozen cells were treated in the same manner for comparison.

### 1.6.3 Cytochrome P450 (CYP450) Activity

HepG2 cells (15k cell/well) were seeded in collagen (12.7 µg/cm$^2$) coated 96 well plates for 24 hours and subsequently freeze/thawed using the optimised protocol described in 1.4. After 24 h of thawing, the cell culture medium was replaced with complete MEM medium supplemented with either 0–50 µM carbamazepine or 0–20 µM rifampicin. The cells were placed in a humidified environment at 37 °C and 5% $CO_2$ for 48 h. To determine CYP450 activity, CYP3A4 and CYP2C9 were measured using the corresponding Promega CYP450 kits (V8792 and V9002). Briefly, the medium was replaced with a culture medium containing a luminogenic CYP substrate, either CYP3A4/Luciferin-IPA (3 µM, 50 µL, 1 h) or CYP2C9/Luciferin-H (100 µM, 50 µL, 3 h). The CYP substrate was also added to empty wells as a background measurement. The culture medium containing the CYP substrate (25 µL) was transferred to an opaque white 96 well plate, and luciferin detection reagent (25 µL) was added for 20 min at RT. Luminescence was measured on a TECAN Spark microplate reader with 1 s integration time. The CYP activity of non-frozen cells was also measured.

## 1.6 High-Throughput Drug Screening

HepG2 cells (15k cell/well) were seeded on collagen coated plates and freeze/thawed using the optimised protocol described in 1.4. Cell culture medium was replaced by cell culture media supplemented with acetaminophen (0–92.6 mM), diclofenac (0–3.4 mM), doxorubicin (0–9.2 mM), metformin (0–310 μM), phenformin (0–97.4 μM) and valproic acid (0–50 mM) and placed in a humidified environment at 37 °C and 5% $CO_2$ for 24 h. The cell culture medium was replaced with 100 μL of resazurin solution, prepared by dissolving 1 resazurin tablet (Scientific Laboratory Supplies, Nottingham, UK) in 50 mL of phenol-free DMEM/F-12 medium supplemented with 10% FBS and sterile filtered. The cells were incubated for 2 h in a humidified environment at 37 °C and 5% $CO_2$. Fluorescence measurements were recorded with excitation and emission at 530/25 nm and 590/30 using a BioTek Synergy HT microplate reader. Wells with resazurin solution alone were also measured for background subtraction and cells untreated with pharmaceutically active compounds were measured as a maximum resazurin reduction value. Percentage cell viability was reported using the fluorescence readings using the following equation:

$$\frac{Sample\ Fluorescence - Background\ Fluorescence}{Maximum\ (Untreated)\ Fluorescence - Background\ Fluorescence} \times 100$$

## 1.7 Primary Hepatocyte Cryopreservation

Mice were bred at the University of Warwick after local AWERB and Home Office approvals (PP3644080). Hepatocytes were isolated from adult (8- to 12-weeks old) male and female C57BL/6NCrl mice livers, via a two-step perfusion process. After cervical dislocation, the peritoneal cavity was open and a 27G needle was inserted into the vena cava. Perfusion was carried out through the vena cava at a flow rate of 6 mL.min$^{-1}$ for 10 mins with buffers warmed

to 42 °C. Perfusion buffer I consisted of HBSS with no $Ca^{2+}$ or $Mg^{2+}$ supplemented with EDTA (0.5 mM) and HEPES (25 mM); the portal vein was cut seconds after observing swelling of the liver with this buffer. Perfusion Buffer II (digestion buffer) consisted of HBSS with $Ca^{2+}$ or $Mg^{2+}$ supplemented with HEPES (25 mM) and Liberase$^{TM}$ (40 μg·mL$^{-1}$). Following digestion, the liver was removed and dissociated in cold suspension buffer (digestion buffer without Liberase$^{TM}$). The cells were passed through a 70 μm filter and centrifuged at 50 g for 2 min at 4 °C. After two washes with plating medium (Williams E supplemented with CM3000 thawing/plating supplement pack), cells were plated in 96 well plate coated with rat tail type I collagen (100 μg·mL$^{-1}$) at a density of 30k cell/well for 4 hours. Other densities used are outlined in specific assays. The medium was exchanged for maintenance medium (Williams E medium supplemented with CM4000 maintenance pack) and the cells were incubated for a further 24 hours. The mouse hepatocytes were cryopreserved using the method described in 1.4. Hornbeam pollen washing water (PWW) was prepared by weighing 0.8 g of hornbeam pollen and suspending it in $H_2O$ for 24 h at 4 °C. The PWW solutions were sterile filtered and mixed 1:1 with Williams E base medium supplemented with 20% FBS and 20% DMSO (final concentration 10%) to produce the cryopreservation formulation. The hepatocyte medium was replaced with 100 μL of the prechilled cryopreservation formulation (4 °C) and the plates were placed on a Corning XT CoolSink 96F and in a −80 °C freezer overnight. Cells were also cryopreserved with this method in a solution containing Williams E base medium supplemented with 10% FBS and 10% DMSO (no pollen) for comparison against the 'gold standard' cryoprotectant alone. Cells were removed from the −80 °C and 150 μL of warm thawing cell medium (37 °C) was added. The cells were placed in the incubator for 12 mins, to allow thawing, and the solutions were replaced with 200 μL of warm maintenance medium (37 °C). The cells were placed in the incubator for a further 24 h. Percentage cell recovery was determined by dissociating cells with trypsin (0.25%) and EDTA (1 mM), staining the cells

with trypan blue (to count membrane intact cells) and comparing cell counts immediately before and 24 h after freeze/thaw.

Gibco$^{TM}$ Mouse (CD-1) Cryopreserved Hepatocytes, Plateable Male (8–12 weeks) (10890041) were purchased from Fisher Scientific (Loughborough, UK) to compare our cryopreservation approach to commercial suspension cryopreserved hepatocytes. Cells were thawed with thawing medium and plated at a density of 30k cell/well in 96-well plates. The medium was replaced with maintenance medium after 4 hours and incubated for a further 24 hours.

## 1.8 Primary Hepatocyte Viability Post-thaw

### 1.8.1 Lactate Dehydrogenase (LDH) Release

Freeze/thaw hepatocytes, produced as described in 1.7, were assessed for the release of LDH. Briefly, to the hepatocyte medium, 10 µL of ultrapure water was added, to determine the release of LDH to the cell culture medium ('Spontaneous LDH Release'), or 10 µL of 10X Lysis Buffer, provided by the CyQUANT™ LDH Cytotoxicity Assay kit (ThermoFisher Scientific,), was added to determine the total LDH content of the cells ('Maximum LDH Release'). The hepatocytes were incubated at 37 °C for 45 min and 50 µL of each sample medium (Spontaneous and Maximum LDH Activity Controls) was added to a new 96-well plate in triplicate (three technical repeats). The Reaction Mixture provided by the CyQUANT™ LDH Cytotoxicity Assay kit was added to each well and mixed by tapping. The plates were incubated at RT for 30 min and 50 µL of stop solution was added. Absorbance measurements were recorded on a BioTek Synergy HT microplate reader at 490 nm and 680 nm (background measurement). Both the Spontaneous and Maximum LDH Release Control Absorbance values were plotted following subtraction of the background absorbance measurement. Non-frozen control cell samples were also analysed following this protocol for comparison.

### 1.8.2 Calcein/ Ethidium Iodide Staining

Freeze/thaw hepatocytes, produced as described in 1.7 but with a density of 15k cell/well, were stained with calcein and ethidium iodide. The cell culture medium was replaced with 100 µL of maintenance medium supplemented with ethidium iodide (2 µM) and calcein (2 µM) and incubated at RT for 40 min. Non-frozen hepatocytes were also stained with ethidium iodide and calcein as a negative control, respectively. Cells were imaged on an Olympus CX41 microscope equipped with a UIS-2 20x/0.45/∞/0−2/FN22 lens using a phase contrast channel and with blue (calcein) and green (ethidium) excitation lasers. Cells were counted using ImageJ. Values were reported as percentage live cells relative to the total number of cells.

### 1.8.3 Resazurin Reduction Metabolic Assay

Freeze/Thaw hepatocytes and Gibco™ Mouse (CD-1), prepared as described in 1.7, were assessed for metabolic activity. The cell culture medium was replaced with 100 µL of resazurin solution, prepared by dissolving 1 resazurin tablet in 50 mL of maintenance medium and sterile filtered. The cells were incubated for 24 h in a humidified environment at 37 °C and 5% $CO_2$. Absorbance measurements were obtained at 570 nm and 600 nm and fluorescence measurements were recorded with excitation and emission at 530/25 nm and 590/30 using a BioTek Synergy HT microplate reader. Wells with resazurin solution alone were also measured for background subtraction. A resazurin reduction assay of non-frozen cells was also completed for comparison. Results were reported as relative resazurin reduction (absorbance) or normalised resazurin reduction (fluorescence).

### 1.8.4 CYP response

Freeze/thaw hepatocytes and Gibco™ Mouse (CD-1) Cryopreserved Hepatocytes, prepared as described in 1.7, were placed in a humidified environment at 37 °C and 5% $CO_2$ for 48 h before CYP testing. To determine CYP450 activity, CYP3A4 and CYP2C9 were measured using the

corresponding Promega CYP450 kits (V8792 and V9002). Briefly, the medium was replaced with maintenance medium supplemented with a luminogenic CYP substrate, either CYP3A4/Luciferin-IPA (3 µM, 50 µL, 1 h) or CYP2C9/Luciferin-H (100 µM, 50 µL, 3 h). The CYP substrate was also added to empty wells as a background measurement. The culture medium containing the CYP substrate (25 µL) was transferred to an opaque white 96 well plate, and luciferin detection reagent (25 µL) was added for 20 min at RT. Luminescence was measured on a TECAN Spark microplate reader with 1 s integration time. The CYP activity of non-frozen cells was also measured.

#### 1.8.5 Urea secretion

Urea measurements for Freeze/thaw hepatocytes and Gibco™ Mouse (CD-1) Cryopreserved Hepatocytes, prepared as described in 1.7, were taken 24 hours post-thaw using the Sigma-Aldrich Urea Assay kit (MAK006). The supernatant of freeze/thaw and non-frozen cells were collected (2 µL) and diluted in Urea assay buffer (48 µL); this was completed four times for each sample. Complete reaction mix was added to two wells (50 µL: Urea assay buffer, 42 µL; Peroxidase substrate, 2 µL; Enzyme mix, 2 µL; Developer, 2 µL; Converting enzyme, 2 µL) and reaction mix without converting enzyme was added to two wells for each sample (50 µL: Urea assay buffer, 44 µL; Peroxidase substrate, 2 µL; Enzyme mix, 2 µL; Developer, 2 µL), to subtract backgrounds generated by ammonium ion, NAD+/NADP+, and pyruvate. The plate was mixed (by pipetting) and incubated for 60 min in a humidified environment at 37 °C and 5% $CO_2$. Absorbance measurements were recorded at 570 nm using a BioTek Synergy HT microplate reader. Values were compared against a urea calibration curve of 0–5 $nmol.well^{-1}$ and reported as $nmol.\mu L^{-1}$.

### 1.8.6 Drug-dose response

The cell culture medium of freeze/thaw hepatocytes, produced in section 1.7, was replaced by maintenance medium supplemented with diclofenac (0–3.4 mM) and metformin (0–77 μM) and placed in a humidified environment at 37 °C and 5% $CO_2$ for 24 h. The cell culture medium was replaced with 100 μL of resazurin solution, prepared by dissolving 1 resazurin tablet (Scientific Laboratory Supplies, Nottingham, UK) in 50 mL of maintenance medium supplemented with 10% FBS and sterile filtered. The cells were incubated for 24 h in a humidified environment at 37 °C and 5% $CO_2$. Fluorescence measurements were recorded with excitation and emission at 530/25 nm and 590/30 using a BioTek Synergy HT microplate reader. Wells with resazurin solution alone were also measured for background subtraction and cells untreated with pharmaceutically active compounds were measured as a maximum resazurin reduction value. Percentage cell viability was reported using the fluorescence readings using the following equation:

$$\frac{Sample\ Fluorescence - Background\ Fluorescence}{Maximum\ (Untreated)\ Fluorescence - Background\ Fluorescence} \text{ x } 100$$

# 2 Results

## 2.1 Screening cryoprotectants

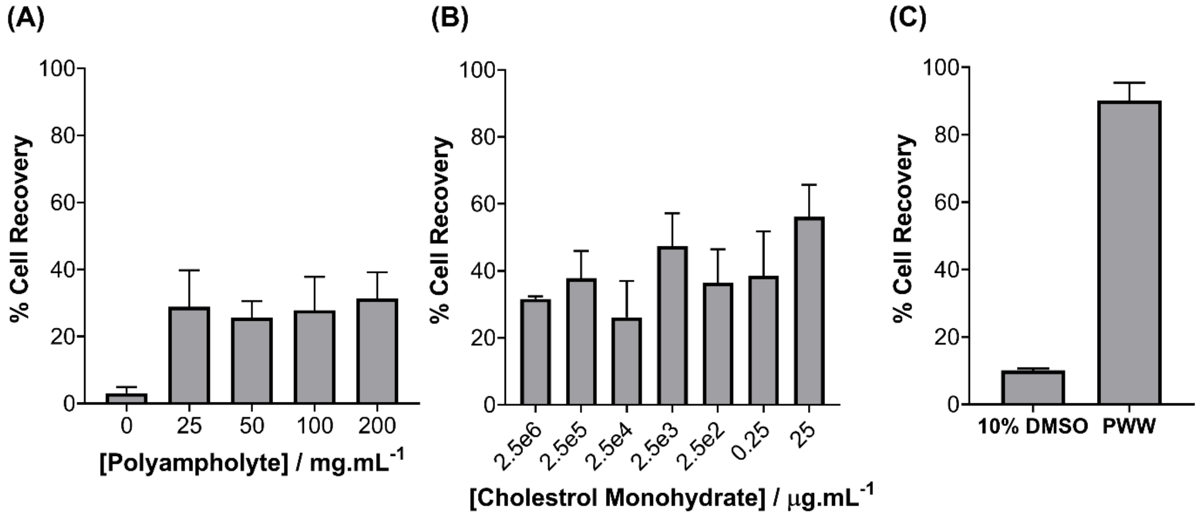


**Figure S1. Screening Cryoprotectants for 96-well plate cryopreservation.** Percentage cell recovery of HepG2 cells cryopreserved with (**A**) polyampholyte (PA, 0–200 mg·mL$^{-1}$), (**B**) Cholesterol monohydrate (2.5e6–25 μg·mL$^{-1}$) and (**C**) PWW supplemented with 10% DMSO and 10% FBS 24 h post-thaw. The data is represented by mean ± SEM of 2 biological repeats and >2 technical repeats (ANOVA, Tukey PostHoc; ns: $p \geq 0.05$, *$p \leq 0.05$, **$p \leq 0.01$).

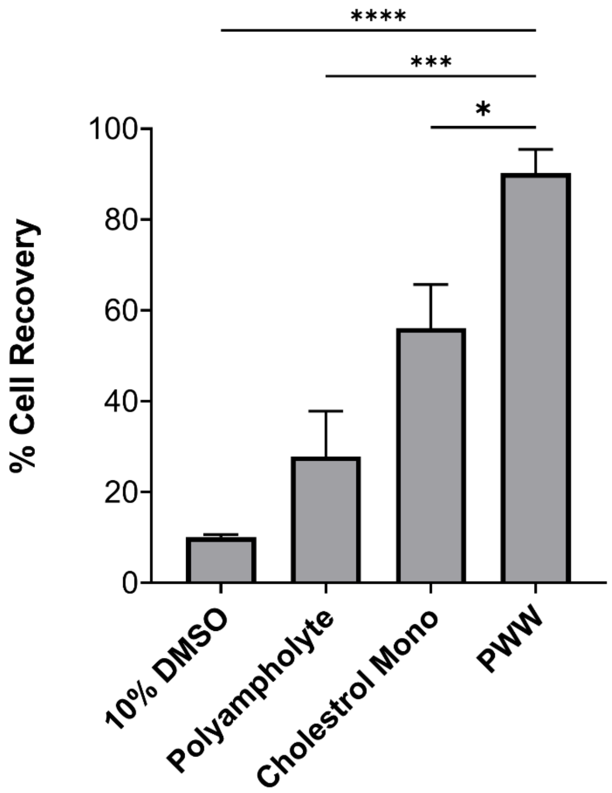


**Figure S2. Highest cell recovery for each cryoprotectant tested.** Post-thaw percentage cell recovery of HepG2 cells cryopreserved with 10% DMSO alone or polyampholyte (100 mg·mL$^{-1}$), Cholesterol monohydrate (25 μg·mL$^{-1}$) and (**C**) PWW supplemented with 10% DMSO. Cell recoveries were determined 24 h post-thaw and the data is represented by mean ± SEM of 2 biological repeats and >2 technical repeats (ANOVA, Tukey PostHoc; *$p \leq 0.05$, ***$p \leq 0.001$, ****$p \leq 0.0001$).

## 2.2 PWW Post-Thaw Cell Recovery and Images

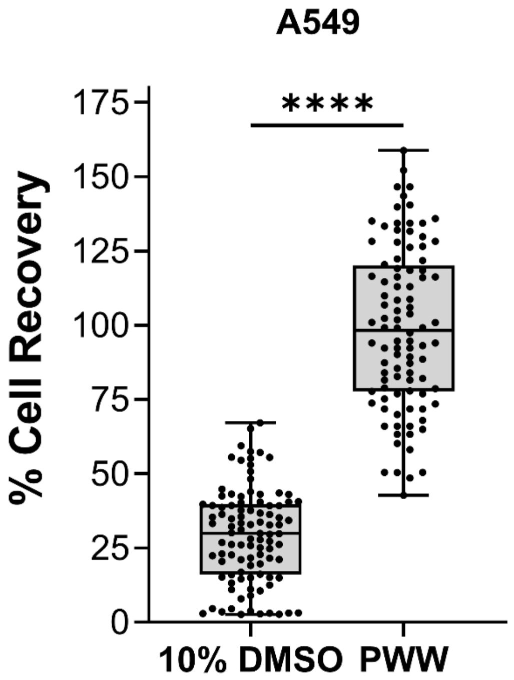


**Figure S3. A549 recovery following freeze/thaw.** Post-thaw percentage cell recovery of A549 cells cryopreserved with 10% DMSO alone or PWW supplemented with 10% DMSO. Cell recoveries were determined 24 h post-thaw and the data is represented by median ± upper/lower quartiles and min/max value of 3 biological repeats and 30 technical repeats (ANOVA, Tukey PostHoc; ****$p \leq 0.0001$).

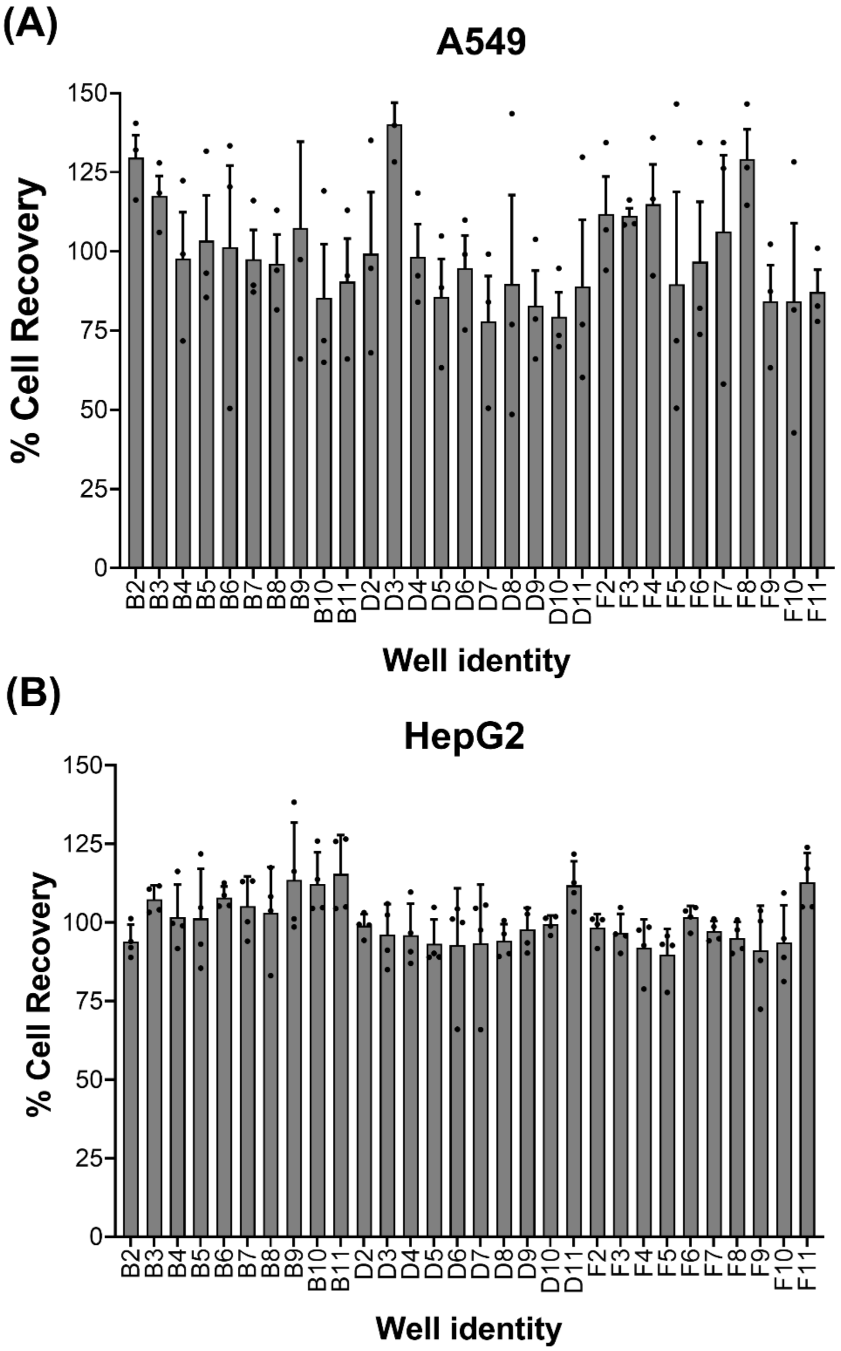


**Figure S4. Well plate variation.** Well-to-well variation in the post-thaw percentage cell recovery of **(A)** A549 and **(B)** HepG2 cells cryopreserved with PWW supplemented with 10% DMSO. Cell recoveries were determined 24 h post-thaw and the data is represented by mean ± SEM of 3 (A549) or 4 (HepG2) biological repeats.

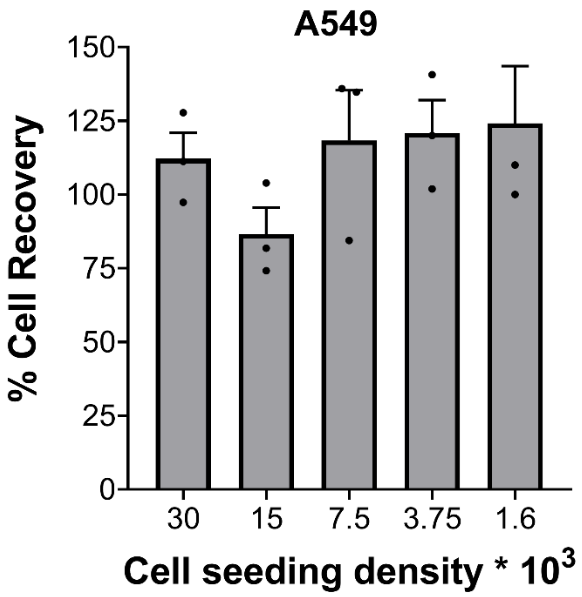


**Figure S5. Cryopreserving cells at different seeding densities.** Post-thaw percentage cell recovery of A549 cells at seeding densities of 1.6 – 30 k cells per well cryopreserved with PWW supplemented with 10% DMSO. Cell recoveries were determined 24 h post-thaw and the data is represented by mean ± SEM of 3 biological repeats.

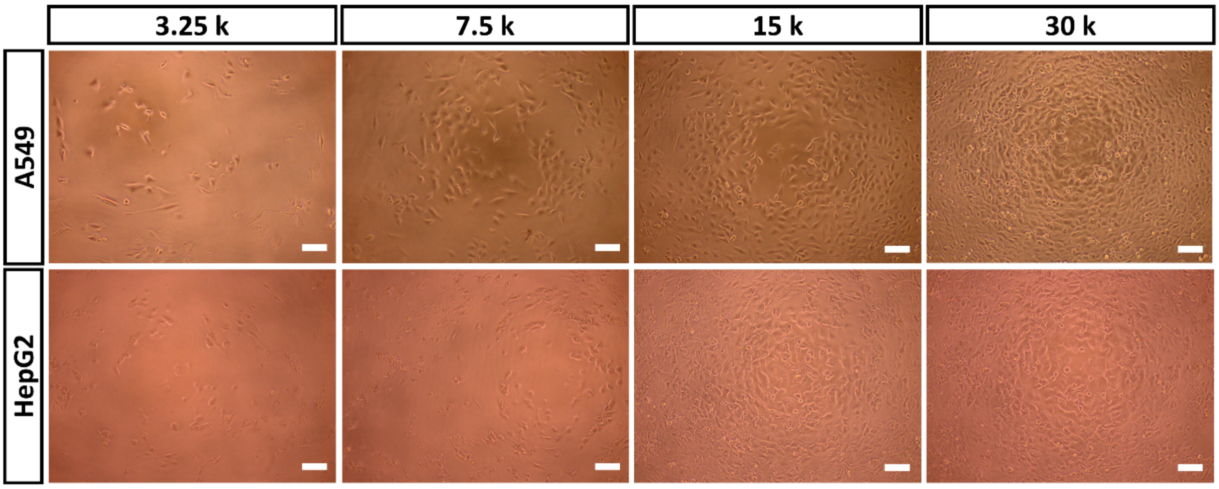


**Figure S6. Post-thaw phase contrast imaging.** Phase contrast images of A549 and HepG2 cells following freeze/thaw at seeding densities of 1.6 – 30 k cells per well. The cells were cryopreserved with PWW supplemented with 10% DMSO. Scale bar = 100 µm.

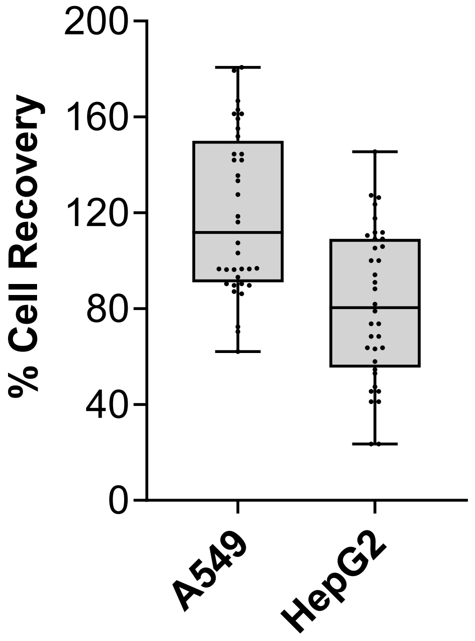


**Figure S7. Long-term storage of A549 and HepG2 cells.** Post-thaw percentage cell recovery of A549 and HepG2 cells (7k cell per well) stored in a -80 °C for over a month. The cells were cryopreserved with PWW supplemented with 10% DMSO.

## 2.3 Cell Viability Measurements

### 2.3.1 Live/Dead

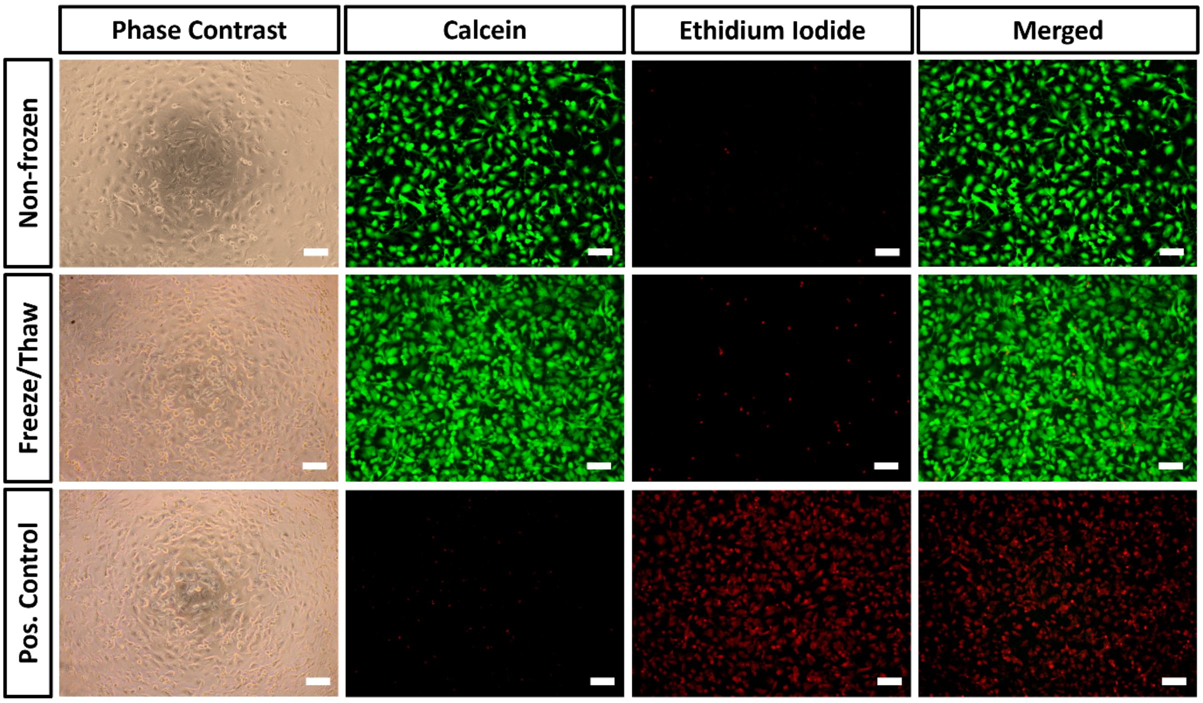


**Figure S8. HepG2 Live/Dead Staining.** Freeze/thaw HepG2 cells were stained with calcein (green, intact membrane) and ethidium iodide (red, damaged membrane) 24 h post-thaw. Non-frozen cells and cells treated with ice cold 70% methanol for 30 mins (positive control) were also stained for comparison. Scale bar = 100 µm.

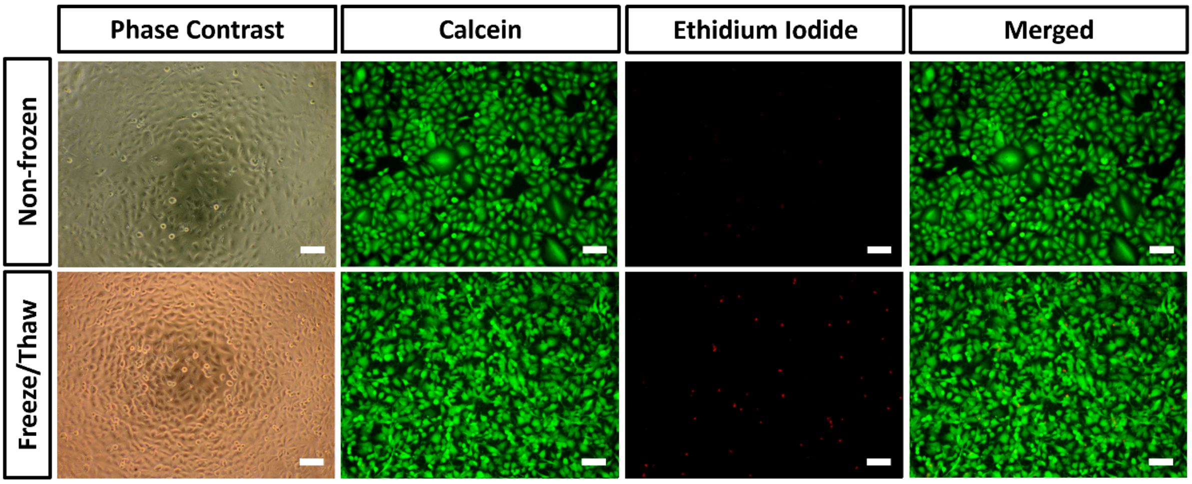


**Figure S9. A549 Live/Dead Staining.** Freeze/thaw A549 cells were stained with calcein (green, intact membrane) and ethidium iodide (red, damaged membrane) 24 h post-thaw. Non-frozen cells were also stained for comparison. Scale bar = 100 µm.

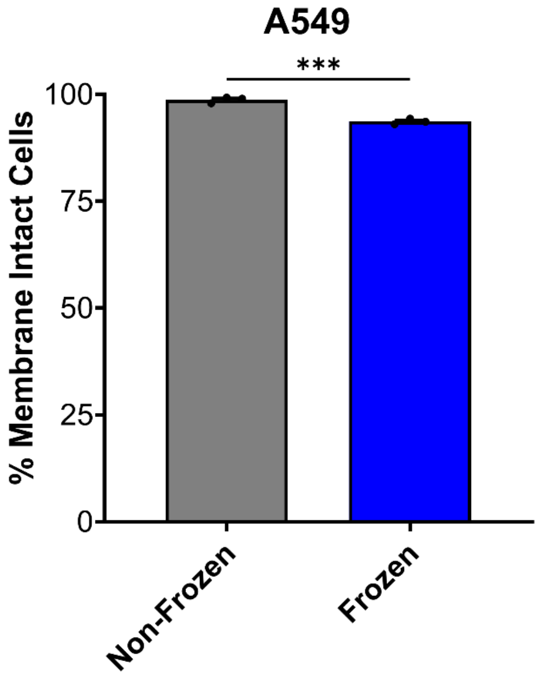


**Figure S10.** Membrane integrity of freeze/thaw cells. The percentage of membrane intact A549 cells from the images in Fig. S8 were determined and reported ± SEM of 3 biological repeats (ANOVA, Tukey PostHoc; ***$p \leq 0.001$).

2.3.2 Caspase-3/-7

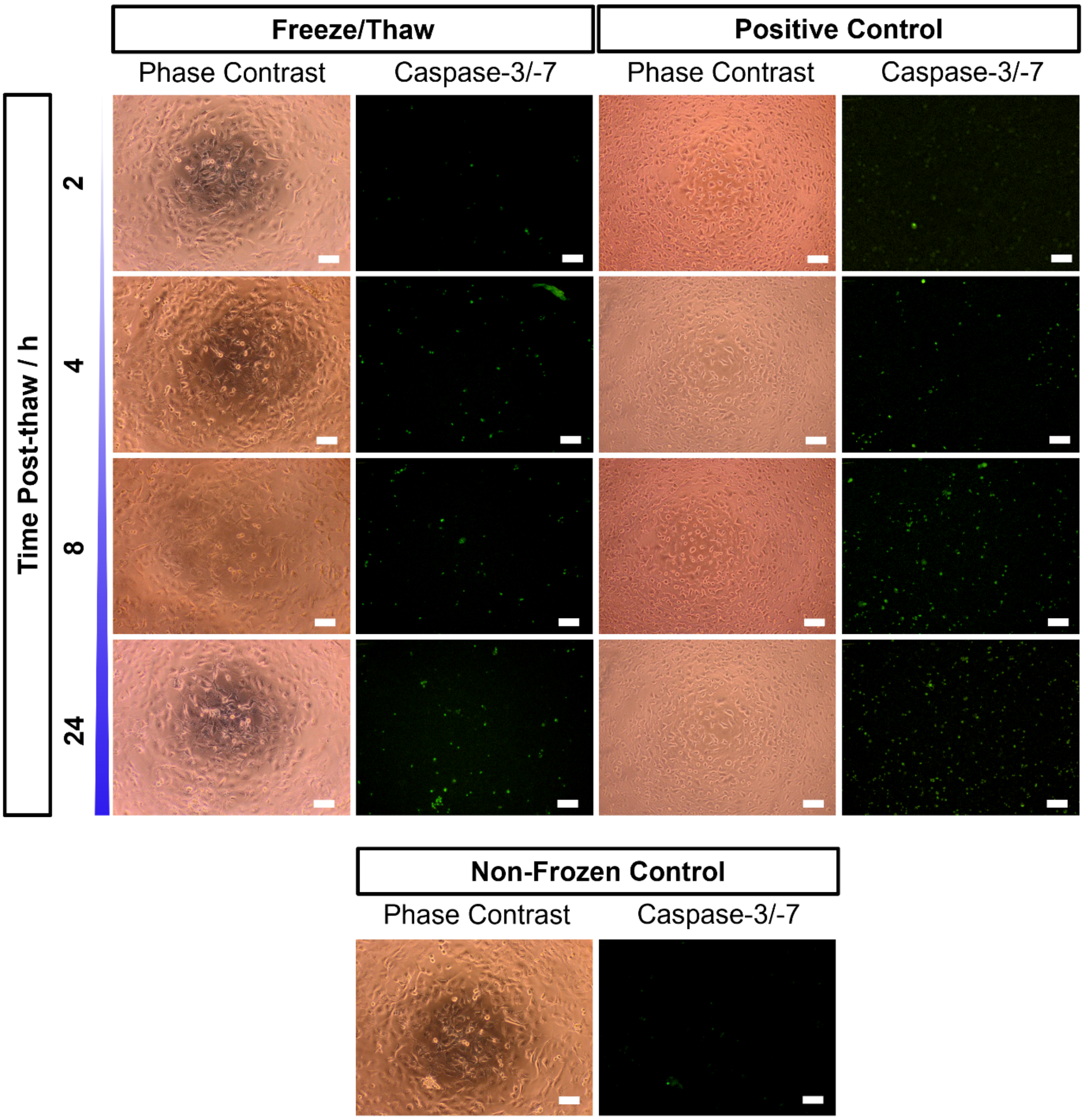


**Figure S11. Real-time caspase-3/-7 activation in freeze/thaw HepG2 cells.** Caspase-3/-7 (green) activation was stained in freeze/thaw HepG2 cells and imaged over 2–24 h post-thaw. A positive control of non-frozen HepG2 cells incubated with staurosporine (2 µM) and non-frozen HepG2 cells were stained for Caspase-3/-7 activation for comparison. Scale bar = 100 µm.

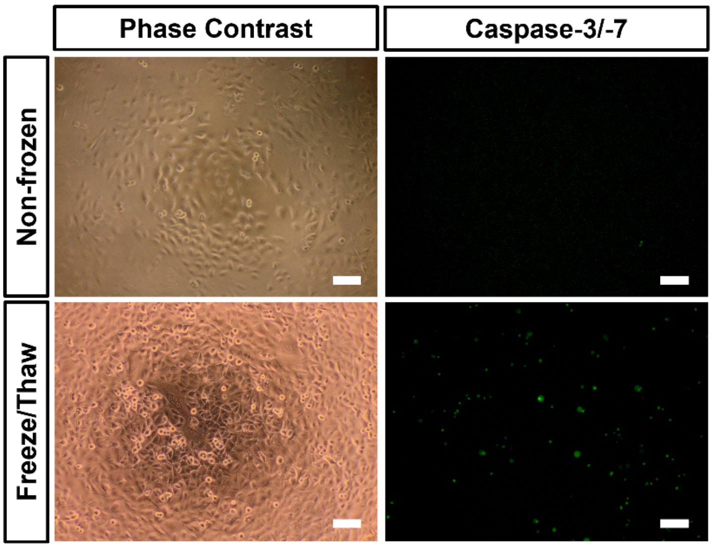


**Figure S12. Caspase-3/-7 activation in freeze/thaw A549 cells.** Caspase-3/-7 (green) activation was stained in freeze/thaw A549 cells and imaged 24 h post-thaw. Non-frozen A549 cells were also stained and imaged for comparison. Scale bar = 100 μm.

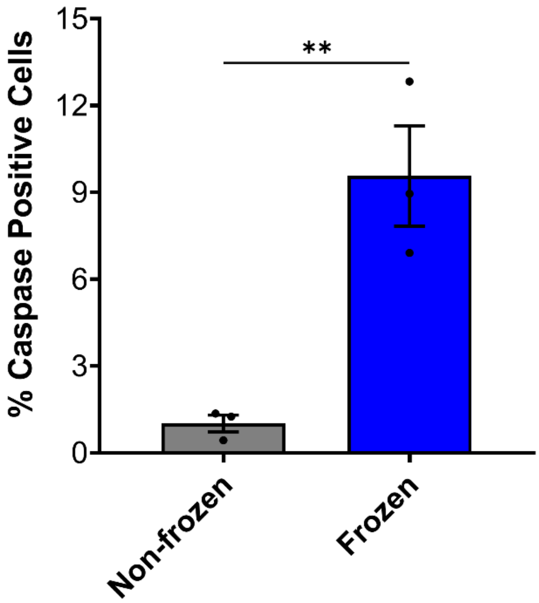


**Figure S13. Post-thaw apoptosis in cryopreserved A549 monolayers**. Percentage caspase-3/-7 positive A549 cells before (non-frozen) and after freeze/thaw. Average percentage caspase-3/-7 positive cells was reported ± SEM of 3 biological and 1 technical repeat.

### 2.3.3 Resazurin Reduction Metabolic Assay

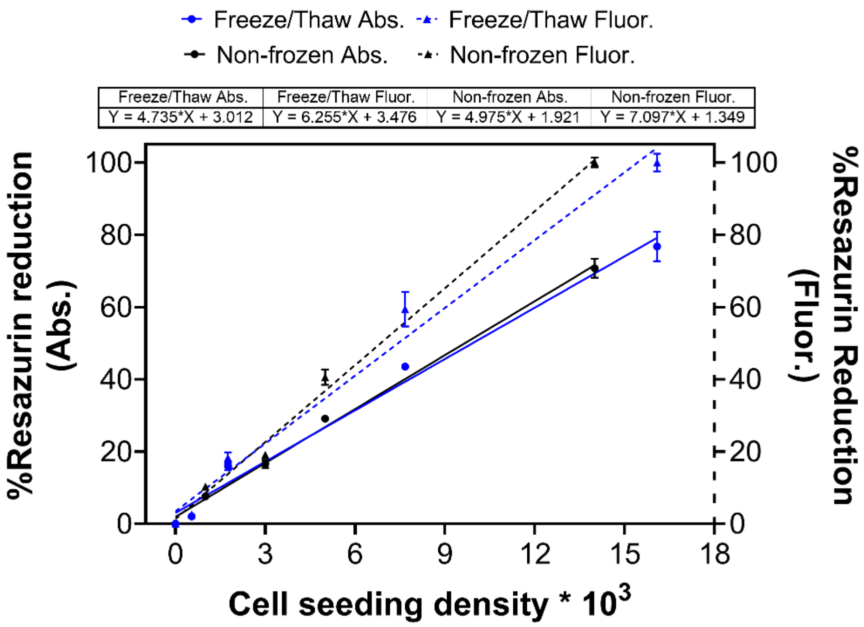


**Figure S14.** Metabolic assessment of freeze/thaw HepG2 cells. HepG2 cells (0–15k cells per well) were cryopreserved with PWW. Frozen cells (**blue**) were thawed and a resazurin reduction assay was completed 24 h post-thaw alongside non-frozen cells (**black**). Resazurin reduction was reported as an average using absorbance (**Solid line**) and fluorescence (**dashed line**) measurements ± SEM of 3 biological repeats and 4 technical repeats.

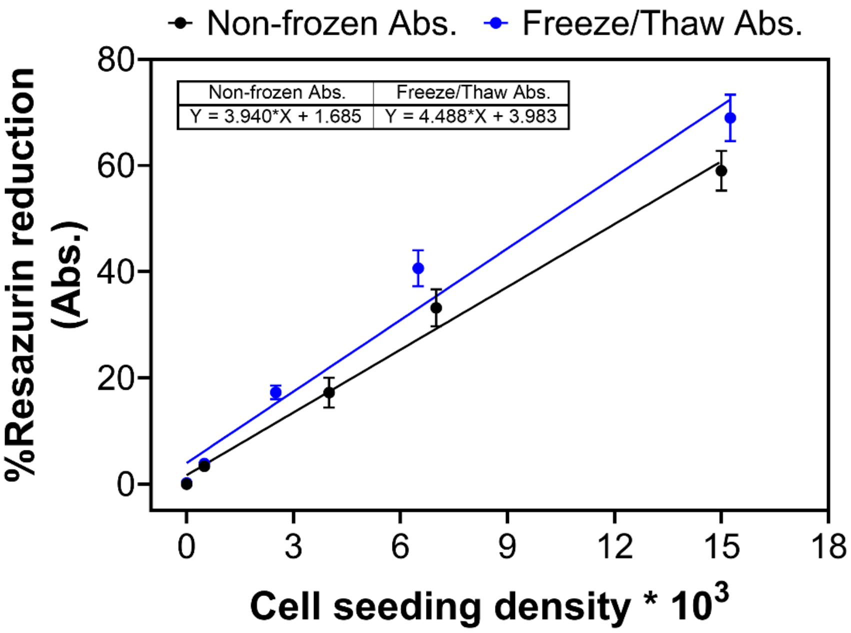


**Figure S15.** Metabolic assessment of freeze/thaw A549 cells. A549 cells (0–15k cells per well) were cryopreserved with PWW. Frozen cells (**blue**) were thawed and a resazurin reduction assay was completed 24 h post-thaw alongside non-frozen cells (**black**). Percentage resazurin reduction was reported as an average ± SEM of 3 biological repeats and 2 technical repeats.

### 2.3.5 Growth Curves

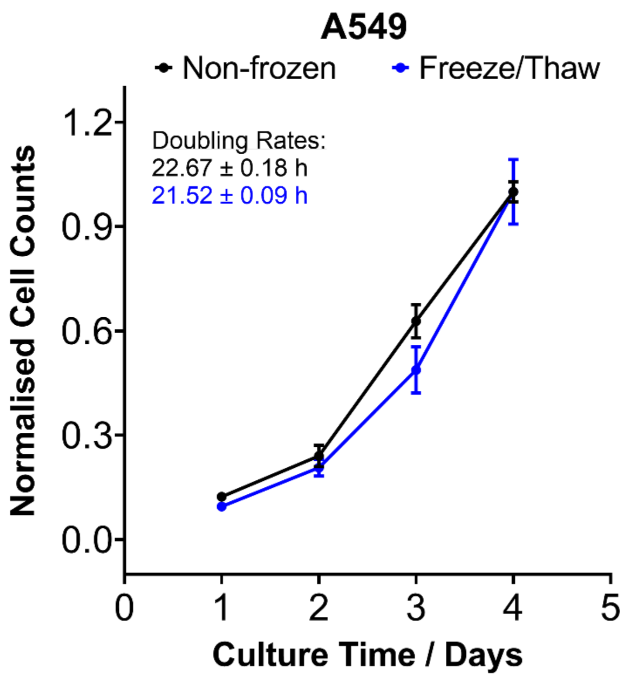


**Figure S16. Proliferation rate after freeze/thaw.** Growth curves for non-frozen (**black**) and freeze/thaw (**blue**) A549 cells. Growth curves were produced by normalising daily counts ± SEM of 3 biological repeats and 2 technical repeats.

## 2.4 Differentiated Hepatic Function in HepG2 Cells

### 2.4.1 Urea secretion

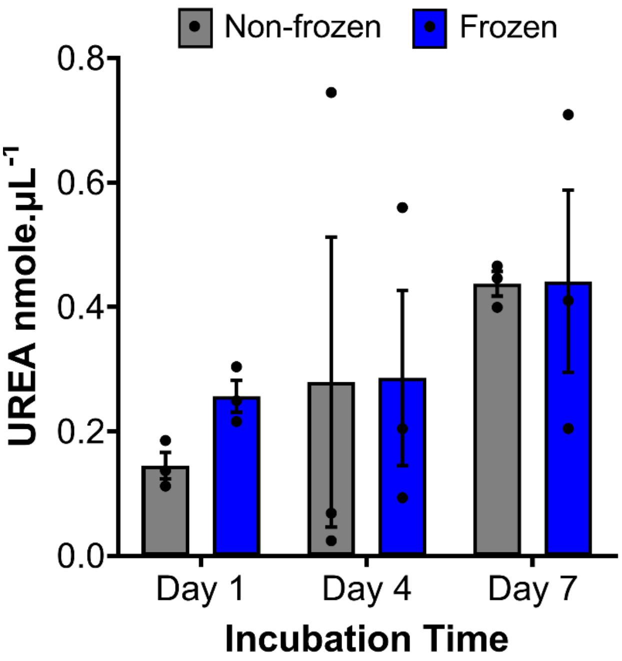


**Figure S17. Urea secretion**. Urea accumulation into culture medium by non-frozen (**grey**) and freeze/thaw (**blue**) HepG2 cells ± SEM of 3 biological repeats (ANOVA, Tukey PostHoc ns: $p \geq 0.05$).

### 2.4.2 Lipid Droplet Accumulation

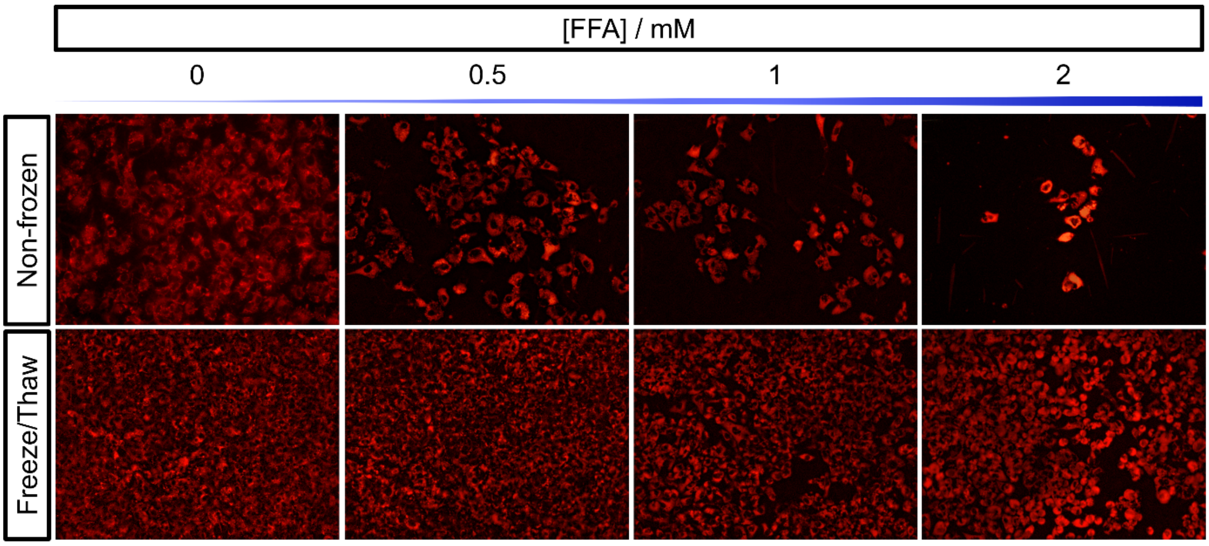


**Figure S18. Imaging fatty acid accumulation in HepG2 cells.** Non-frozen and frozen cells were incubated with a free fatty acid (FFA) solution (1:1 ratio of sodium oleate and sodium palmitate, 0 – 2 μM) for 24 h and stained with Nile red to identify lipid droplets.

2.4.3 CYP Activity Inducers

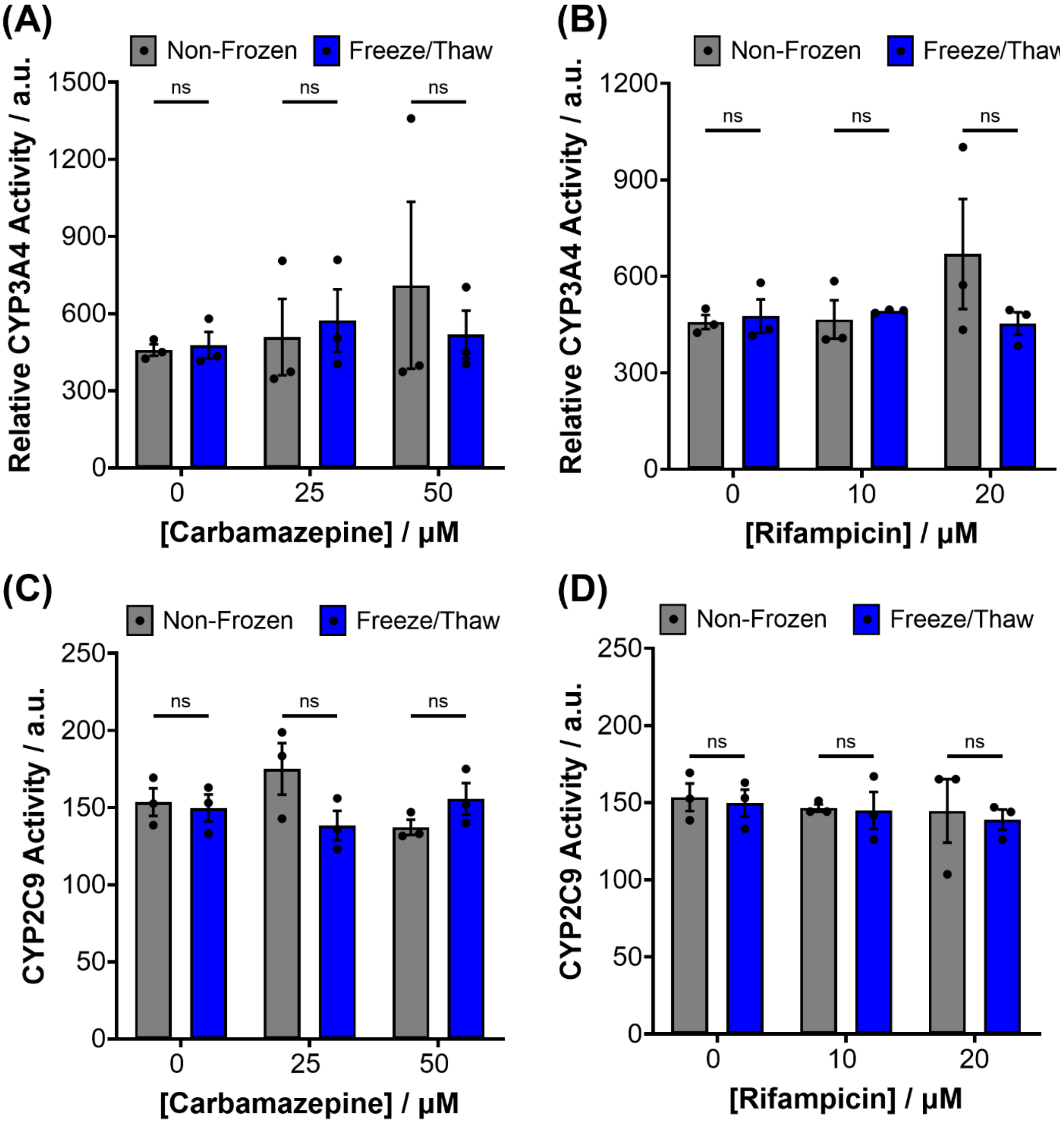


**Figure S19. Cytochrome P450 (CYP450) activity of non-frozen and freeze/thaw cells after incubation with inducers.** (**A**, **B**) CYP3A4 and (**C**, **D**) CYP2C9 activity of non-frozen and freeze-thaw (24 h post-thaw) HepG2 cells after incubating with (**A**, **C**) carbamazepine (0–50 µM) and **(E, D)** rifampicin (0–20 µM) for 48 h. Results are reported as relative CYP activity ± SEM of 3 biological repeats (ANOVA, Tukey PostHoc ns: $p \geq 0.05$).

## 2.5 Freeze/Thaw Primary Hepatocyte Recovery, Viability and Functional Assays

### 2.5.1 ATP

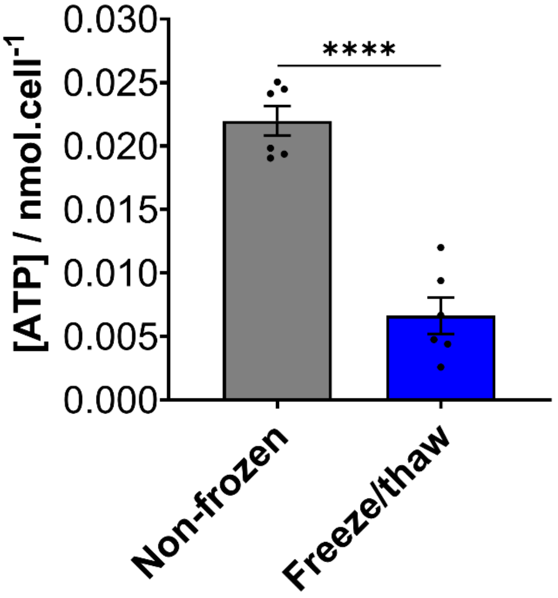


**Figure S20.** ATP content of non-frozen (gray) and freeze/thaw (blue) adherent hepatocytes ± SEM of 3 biological and 2 technical repeats.

2.5.2 Post-Thaw Phase Contrast Images

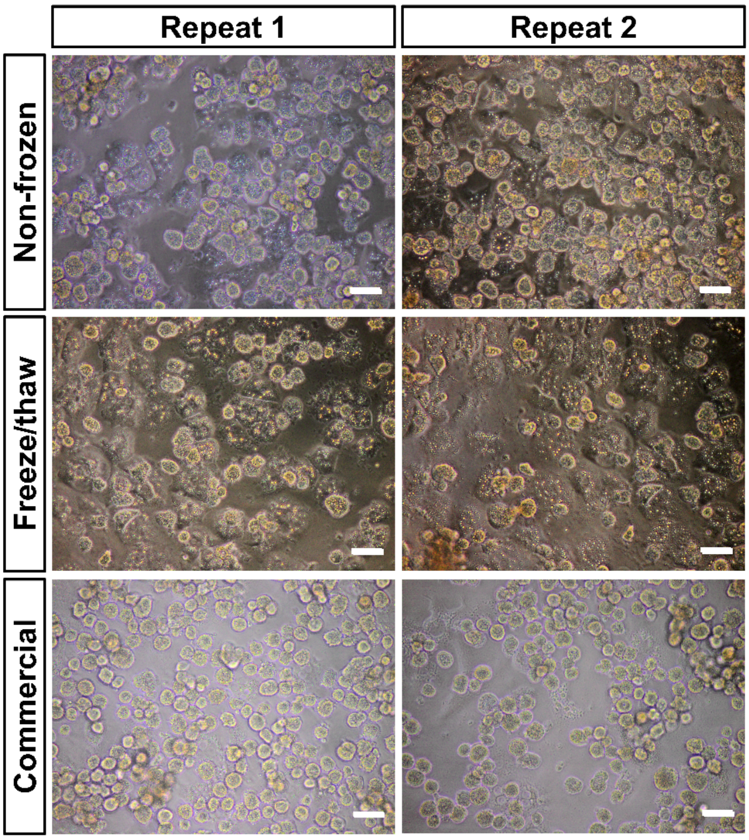


**Figure S21.** Phase contrast images of non-frozen and freeze/thaw adherent hepatocytes (cryopreserved with PWW and 10% DMSO) and suspension cryopreserved hepatocytes purchased from a commercial supplier. Scale bar: x20 = 50 µm.

2.5.3 Calcein/Ethidium Iodide staining

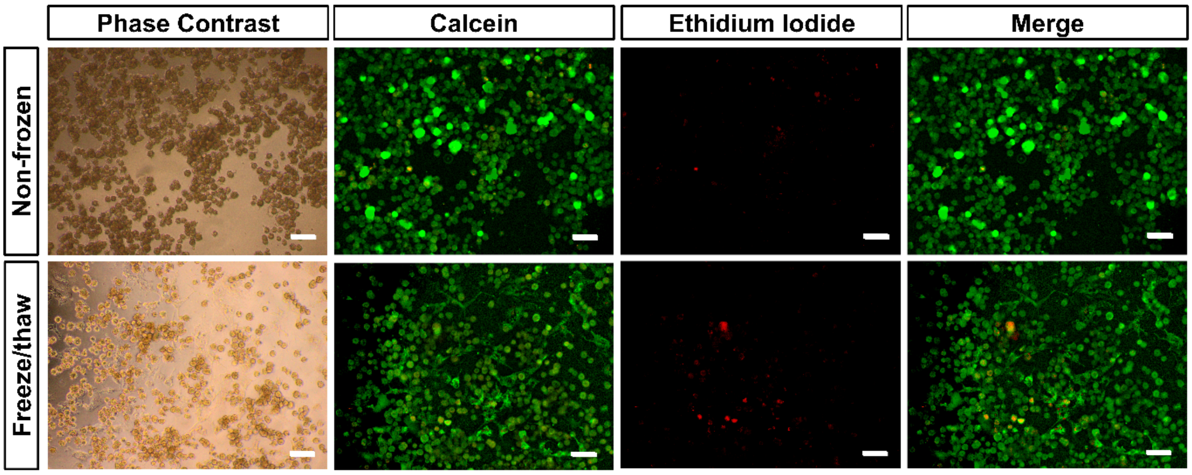


**Figure S22. Primary mouse hepatocyte live/dead staining.** Freeze/thaw primary hepatocytes cells were stained with calcein (green, intact membrane) and ethidium iodide (red, damaged membrane) 24 h post-thaw. Non-frozen cells were also stained for comparison. Scale bar = 100 μm.

## 3.0 References


1. Tomás, R. M. F., Bissoyi, A., Congdon, T. R. & Gibson, M. I. Assay-ready Cryopreserved Cell Monolayers Enabled by Macromolecular Cryoprotectants. *Biomacromolecules* **23**, 3948–3959 (2022).
2. Garti, N., Karpuj, L. & Sarig, S. Correlation between crystal habit and the composition of solvated and nonsolvated cholesterol crystals. *J Lipid Res* **22**, 785–791 (1981).
